# Topographic and climatic controls of peatland distribution on the Tibetan Plateau

**DOI:** 10.1038/s41598-023-39699-x

**Published:** 2023-09-08

**Authors:** Jingjing Sun, Angela Gallego-Sala, Zicheng Yu

**Affiliations:** 1 Key Laboratory of Geographical Processes and Ecological Security in Changbai Mountains (Ministry of Education), School of Geographical Sciences, Northeast Normal University, Changchun, Jilin 130024 China; 2 Geography Department, College of Life and Environmental Sciences, University of Exeter, Exeter, Devon EX4 4QE UK; 3 State Key Laboratory of Black Soils Conservation and Utilization, Northeast Institute of Geography and Agroecology, Chinese Academy of Sciences, Changchun, Jilin 130102 China

**Keywords:** Wetlands ecology, Environmental impact

## Abstract

The Tibetan Plateau (TP) hosts a variety of mountain peatlands that are sensitive to the amplified warming in this region. However, we still lack a basic understanding of environmental and climatic factors controlling peatland distribution in the region. Here we use a bioclimatic envelope model (PeatStash) and environmental analysis that utilise three peatland datasets—(a) the well-studied Zoige peatland complex, (b) a literature-based dataset of TP peatlands sites, and (c) an existing global peatland map (PEATMAP)—to investigate major drivers of peatland distribution in the TP. The Zoige peatland complex is defined by gentle slopes (< 2°), mean annual temperature at 0–2 °C, and soil moisture index > 1.7, much narrower thresholds than those stemming from PEATMAP. Using these narrower thresholds to predict future changes, we found that the Zoige peatland complex will shrink greatly under full-range future warming scenarios (both SSP1–2.6 and SSP5–8.5). Modelling peatland distribution in the entire TP remains challenging because accurate environmental and climate data at high resolution and a reliable peatland distribution map are still lacking. Improved peatland mapping supported by ground-truthing is necessary to understand drivers of peatland distribution, assess carbon storage and other ecosystem services, and predict the TP’s peatlands fate under climate change.

## Introduction

Peatlands are an important component of the global carbon (C) cycle, and there are concerns as to the future of this vast carbon stock under global warming climate predictions^1,2^. Although peatlands occupy only 3% of the global land area, mainly distributed in the boreal and subarctic zones of the Northern Hemisphere, they contain about 550 Gt C^2,3^, roughly equivalent to 25% of the global soil carbon stock^4^. Peatlands are sensitive to climate change, and the peat accumulation is directly affected by local climate and topography^5^. However, not only changes to vertical accumulation, but also shrinkage or expansion of peatland extent will determine whether peatlands become overall a sink or a source of carbon, thus affecting the global carbon cycle^6,7^.

Understanding the current distribution of peatlands is therefore critical to our ability to predict the fate of peatlands and their carbon in a changing climate. Although northern peatlands are abundant above the 45°N latitude, there are large differences among different peatland regions in annual temperature and precipitation, suggesting that there are complex climatic controls^8,9^ and that different carbon dynamics may be at play in these different peatland types^7^. The mean annual temperature of northern peatlands ranges from − 12 to + 5 °C, while the mean annual precipitation is between 200 and 1000 mm^10^. In addition to climate, peatland extent is also controlled by topography as this in turn ultimately controls what parts of the landscape are wetter or drier^11^. Therefore, a good understanding of the current climatic and environmental space of peatlands is necessary if we are to make predictions of future peatland distribution and carbon dynamics under a changing climate.

Bioclimatic envelope models can provide a first approximation to the change to species or ecosystem habitats in a changing climate and may be able to highlight ecosystems at risk^12,13^. Such models indicate, for example, that all of Fennoscandia will become climatically unsuitable for permafrost peatlands by 2040 AD^14^ and the British blanket peatland will gradually retreat towards the north and the west of the British Isles^13^. Most bioclimatic envelope model studies focus on North American and European peatlands^13–15^ where there are extensive areas covered with peatland complexes of a single type, and where the topography is relatively simple. In contrast, peatlands in mountains or plateaus are less well studied. The areas covered by individual peatlands in mountain areas tend to be small and the environmental factors controlling their extent are more complex, because the supporting hydrological characteristics can change at small spatial scales owing to variations in topography, geology, and geochemistry^16^.

**Figure 1:**
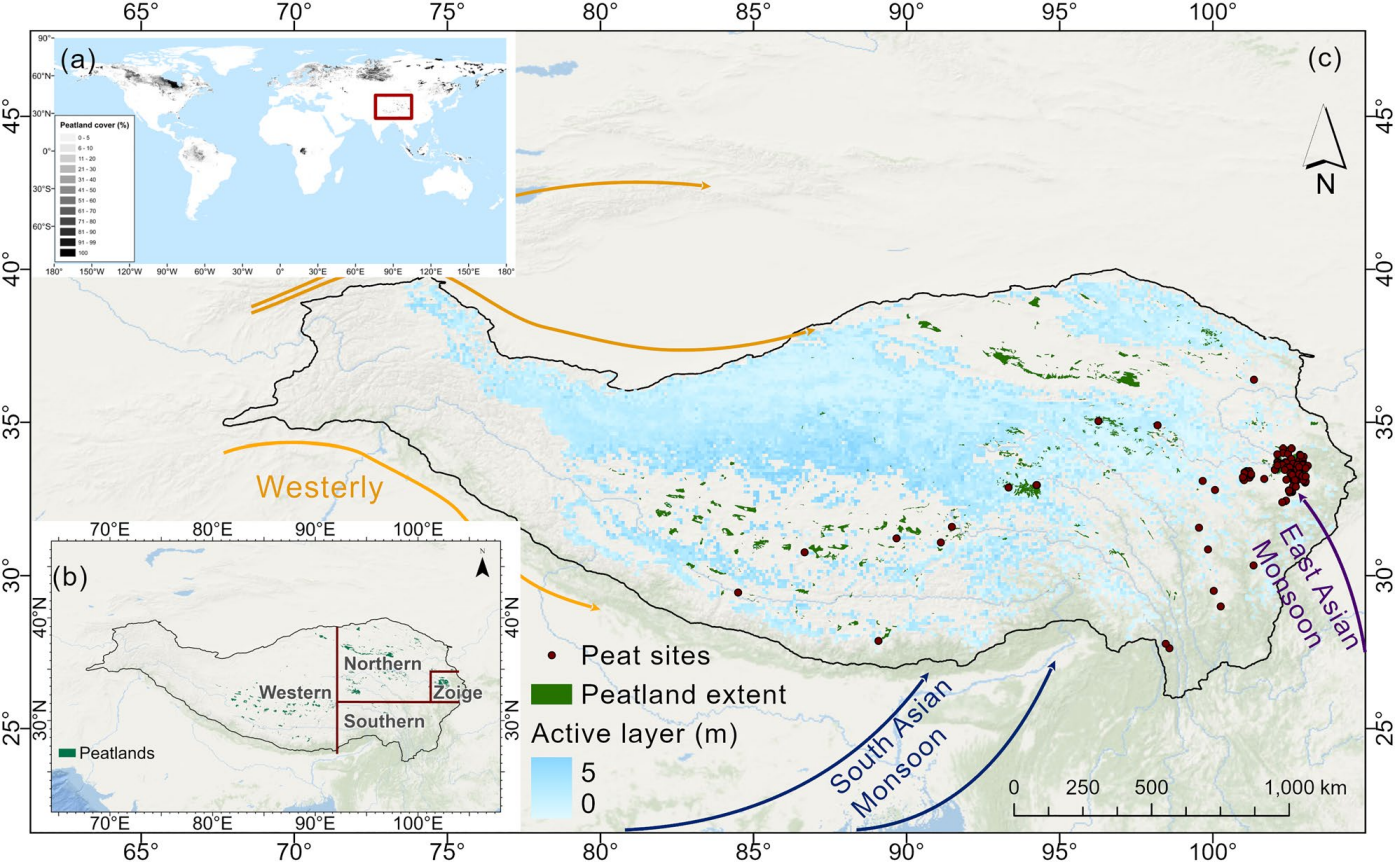
Regional setting and topography of the Tibetan Plateau. (**a**) The global peatland distribution derived from PEATMAP, red box showing the region in (**c**)^20^; (**b**) the peatland distribution (green filled polygons) extracted from PEATMAP, the TP boundary (black line) and the sub-regions considered in the environmental space analysis; and (**c**) the peatland distribution (green polygons extracted from PEATMAP) and the peat site locations (red dots) on the Tibetan Plateau, with the active layer thickness (ALT in m) baseline (the period 2000–2015)^54^. Arrows show dominant atmospheric circulation systems influencing the TP.

High elevation (> 2000 m asl) mountain or plateau peatlands are widely distributed in the North and South American Cordilleras, the Rift Mountains in eastern Africa, and the Tibetan Plateau (TP). These are also areas where climate warming is amplified, especially in the TP, where the rate of warming exceeds those for the Northern Hemisphere and the same latitudinal zone for the same time period^17^. These peatlands constitute a rare habitat and, in some instances, provide multiple ecosystem services to communities living downstream. The TP (Fig. 1) is a complex mountain system also known as the world’s Third Pole, that hosts approximately half (~ 5000 km^2^) of the total peatland area in China^18^. It has accumulated approximately 1.49 Pg of carbon in peat, which accounts for 68% of total peatland C storage in China^19^, although this estimate remains uncertain. The drivers for the development and the expansion of peatlands in the TP are still unclear, but they are likely to be sensitive to climate change and human disturbance.

In this study, we aim (1) to establish the modern environmental space of the TP peatlands; (2) to understand the environmental factors of the present distribution of the well-studied and better mapped Zoige peatland complex in the northeastern TP; (3) to use PeatStash climate envelope model to predict how the distribution is likely to change with future climate change; and (4) to point out the remaining challenges associated with modelling the TP mountain peatlands and future research directions.

## Results

### Tibetan Plateau peatland environmental space

**Figure 2:**
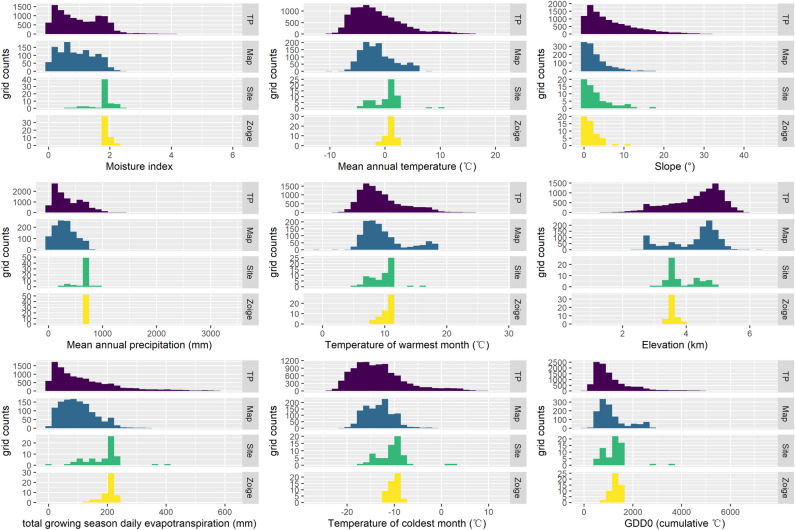
Environmental and climate space. Frequency grids counts of the whole TP landmass (TP, purple, a total of 10,658 grids), peatlands grids in the TP derived from PEATMAP (Map, blue, 1305 grids), literature-based TP peatlands sites in the TP (Site, green, 65 grids), and the Zoige peatland complex (Zoige, yellow, 52 grids).

The present-day distribution of peatlands in the TP (Fig. 1) is the synaptic result of climate and topography. There are large gradients both in temperature and precipitation from the southeast to the northwest of the entire TP land area (Fig. 2). The mean annual temperature (MAT) ranges from − 12 to 16 °C and mean annual precipitation (MAP) ranges from 20 to about 2000 mm (https://www.tibetol.cn/html/2013/gy_0513/968.html). Slopes range from 0° to 30°, as this is a mountainous environment, and elevation often reaches more than 3000 m. In contrast, the peatlands of the TP cover a much narrower environmental and climatic space. There are notable differences among the range of threshold values defining: (1) all the TP peatlands captured in the PEATMAP^20^; (2) the Zoige peatland complex, a peatland-dominated region that is well studied, and for which we therefore have high confidence in terms of peatland distribution; and (3) a dataset of the literature-based peatlands study sites in the TP. The environmental space of peatlands captured by PEATMAP is similar to the environmental space of the whole TP region. For example, PEATMAP shows that peatlands exist in areas with extremely low precipitation and MI (as low as 30 mm in MAP and 0.1 in moisture index (MI)), where such dry conditions should have inhibited peatland formation and persistence^21^. However, the Zoige peatland complex and the literature-based TP peatlands sites present a range of MI values from 1.7 to 2.4, corresponding to values commonly found for other peatlands regions in the world^1^. On the other hand, MAT thresholds for peatlands in the Zoige peatland complex region is between 0 and 2 °C, and also the literature-based TP peatlands sites are distributed in regions with MAT between 1 and 2 °C, so both narrower than the temperature range for TP peatlands shown by PEATMAP (− 5 °C and 2 °C). Finally, peatlands unsurprisingly tend to favour flatter regions that reduce runoff and allow waterlogging. The Zoige peatlands, for example, are distributed in areas with a slope less than 5°, but PEATMAP predicts peatlands existing on slopes of up to 10°; this steep slope is unlikely to be conducive to the water ponding that is necessary to maintain high water tables for peatland development. These findings highlight limitations of the PEATMAP predictions in the TP and the need for ground truthing of predictive maps such as this one.

### PeatStash model performance

**Figure 3:**
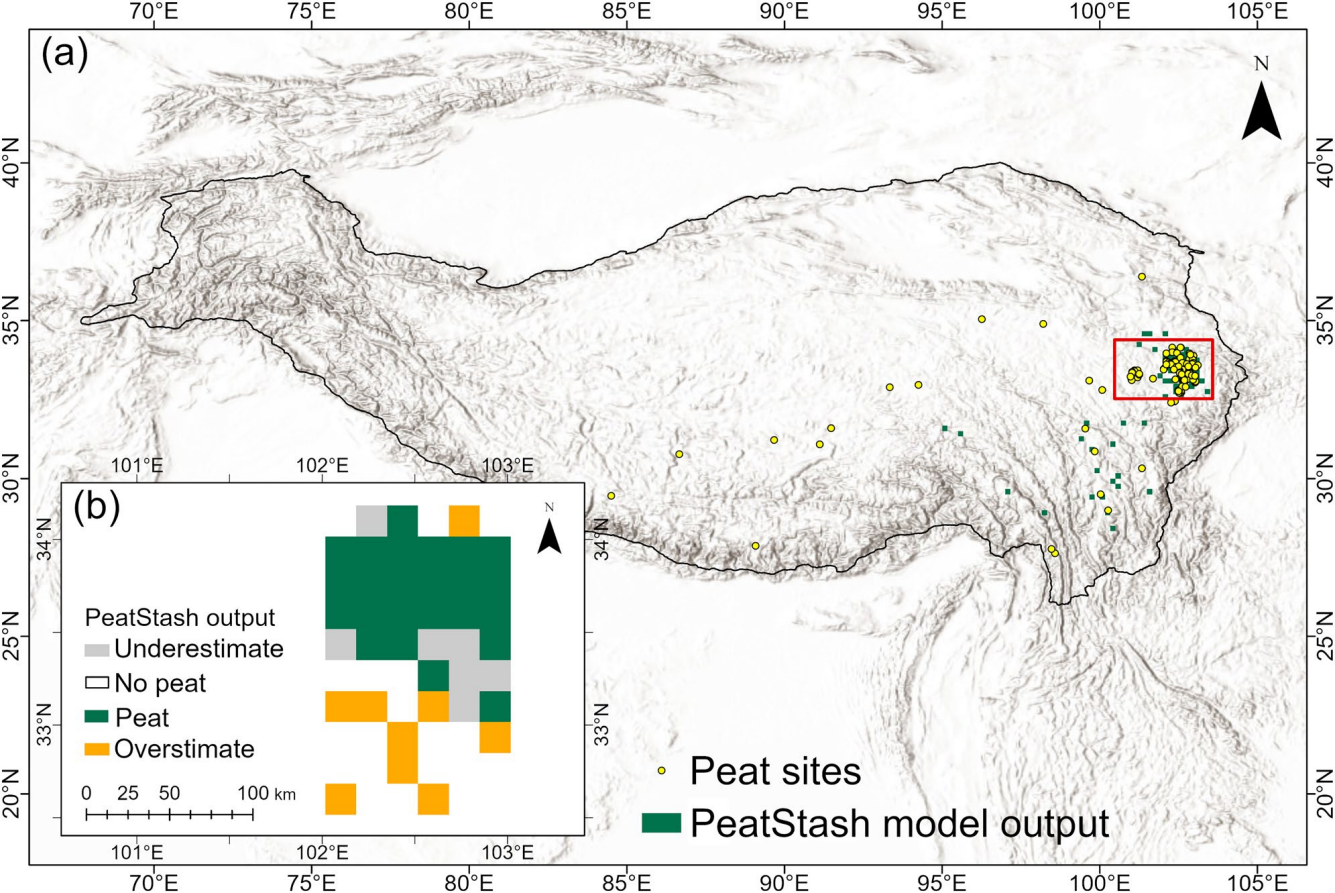
PeatStash present-day output map. (**a**) The output with the narrow threshold (green squares), the literature-based TP peatlands sites (yellow dots), and the Zoige peatland complex region (red rectangle) as shown in (**b**). (**b**) The output of the Zoige peatland complex. Grey areas represent underestimates by the model, where PeatStash predicts absence but PEATMAP shows presence; white areas represent non-peat grids in both PEATMAP and PeatStash model output; green represents areas where both PEATMAP and the PeatStash model predict peat; orange areas represent areas where the Peatstash model predicts peat but there is none in PEATMAP.

**Figure 4:**
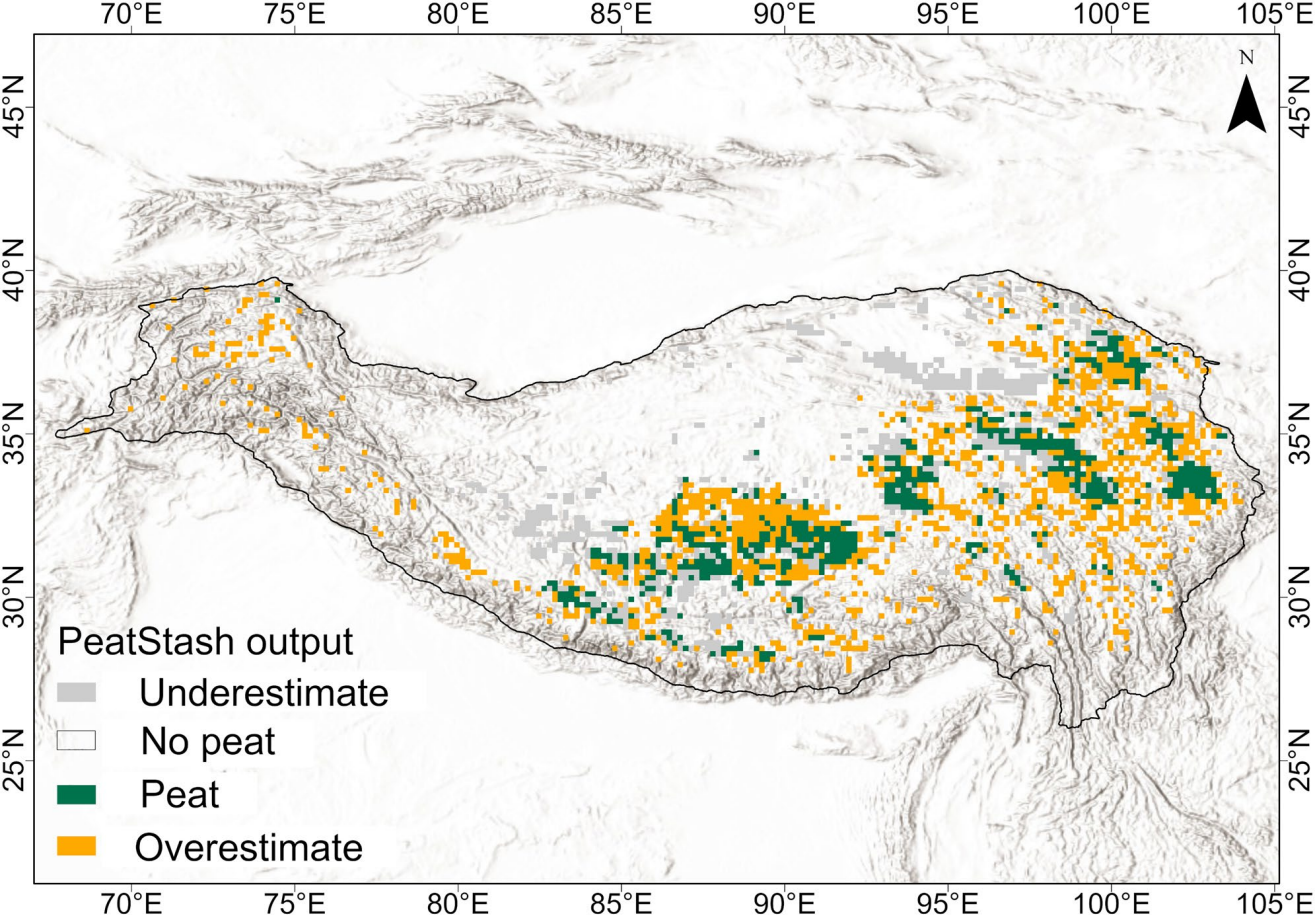
PeatStash present-day output map for the whole TP (wider threshold). Grey areas represent grids where PeatStash predicts no peat but peat is present in PEATMAP; white areas represent non-peat grids in both PEATMAP and PeatStash model output; green represents areas where both PEATMAP and the PeatStash model predict peat; orange areas represent areas where the PeatStash models predicts peat but there is none in PEATMAP.

We set two different thresholds corresponding to the two peatland datasets: (1) a narrow threshold to represent the Zoige peatland complex (this includes most of the known literature-based TP peatlands sites, so the thresholds do not vary whether we include the literature-based TP peatlands sites or not) and (2) a wide threshold to represent the wider TP peatlands as shown in PEATMAP (Table 1). Our final PeatStash model fitted using modern climate data from the CRU CL v. 2.0 climatologies (see Supplementary Fig. S1 online) is able to predict the present environmental and climatic space suitable for peatlands in the TP with a good performance when using the narrow thresholds for the Zoige peatland complex (Fig. 3), but the performance is poorer when using the wider threshold for all the TP peatlands as shown in PEATMAP (Fig. 4). Present-day distribution of the Zoige peatland complex—where the peatland extent is well constrained by observations—was well captured by model predictions that display high sensitivity (0.69), accuracy (0.85), and Kappa statistics (0.65) (Table 2). Meanwhile, model predictions attempting to capture the present distribution of all TP peatlands according to the PEATMAP distribution display poor statistical performance: sensitivity = 0.48, accuracy = 0.80, and Kappa statistics = 0.28 (Table 2). Even though accuracy and sensitivity seem acceptable, this is only a construct of the type of data we are handling. Only 12.7% of the grid cells are peatlands, which means over 85% are non-peat grid areas, that is, even if we were to predict that all of the TP is covered in non-peat grid cells, the accuracy would still be up to 0.85, but it would nonetheless be meaningless. On the contrary, Kappa statistics—defined as the ratio between the observed agreement minus expected agreement over 1 (see methods)—is a better metric for model performance in this case, as both right and wrong grids are considered in the calculation of this quantity.

**Table 1:** Climate and environmental thresholds used to describe the bioclimatic envelope of TP peatlands in the PeatStash model.

Threshold	MI	MAT (°C)	Slope (°)	ALT (m)
Wide threshold for the entire TP				
Upper	ND	2	5	2.3
Lower	0.5	− 5	0	ND
Narrow threshold for Zoige peatlands complex				
Upper	ND	2	2	ND
Lower	1.7	0	ND	ND

*MI* soil moisture index, *MAT* mean annual temperature, *ALT* active layer thickness, *ND* not determined.

**Table 2:** Model sensitivity, accuracy and Kappa statistics when modelling the Zoige peatland complex and the wider TP peatlands using PeatStash.

Region	Sensitivity	Accuracy	Kappa	Number of grid cells
Zoige peatlands complex	0.69	0.85	0.65	65
Entire TP	0.48	0.80	0.28	10,658

### Future environmental and climate space on the TP and changes to peatland distribution

**Figure 5:**
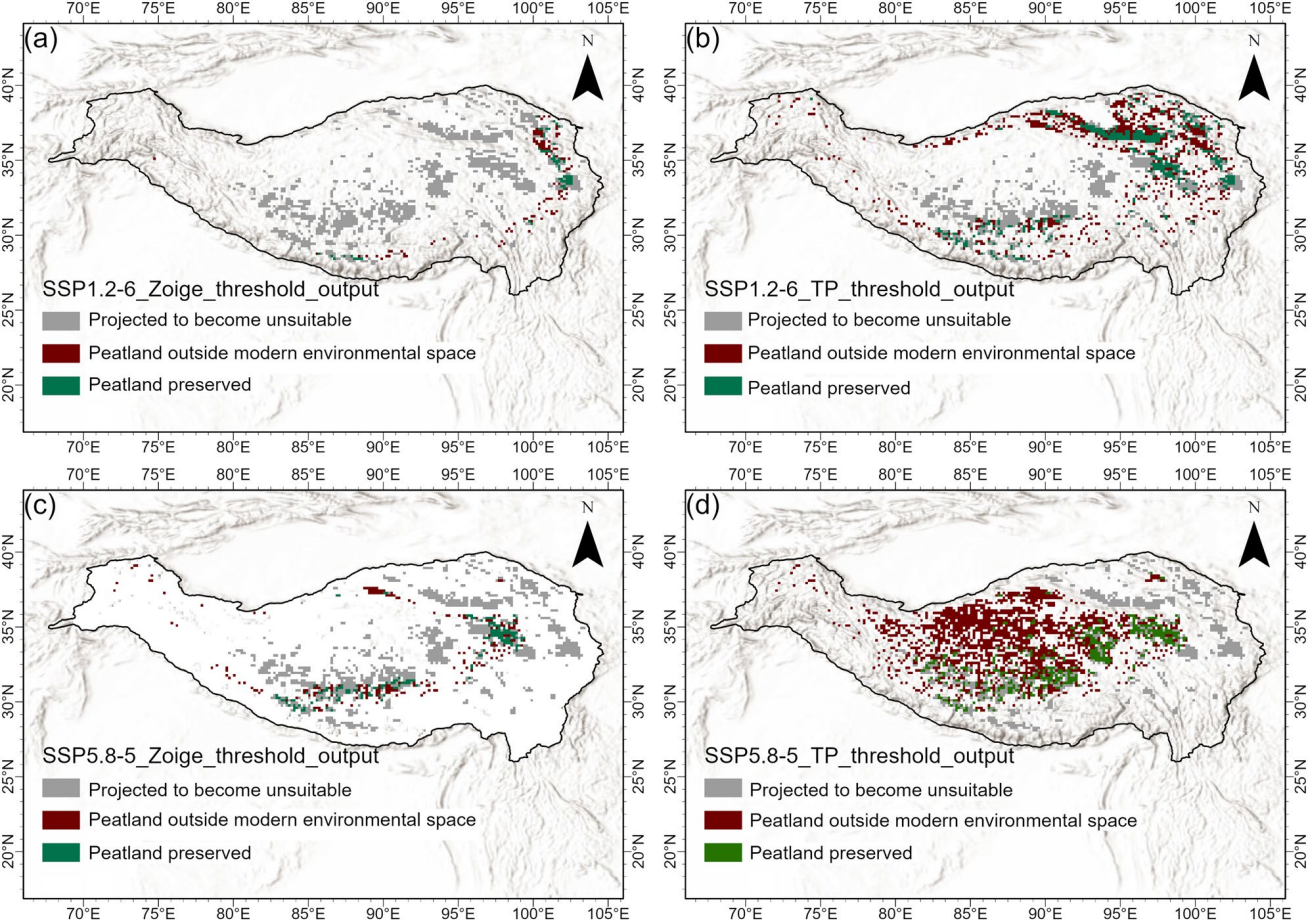
The future peatland distribution on the Tibetan Plateau. (**a**) and (**c**) show the narrow threshold for Zoige peatlands and (**b**) and (**d**) show the wide threshold for the whole TP peatlands from the mean CMIP6 climate model results in 2100 AD. The climate simulations used reflect the two scenarios of Shared Socioeconomic Pathway (SSP) described in the IPCC Sixth Assessment Report: SSP1–2.6 (**a**,**b**), and SSP5–8.5 (**c**,**d**).

We used the climatic thresholds derived from the Zoige peatland complex—since they are likely to better capture the climate necessary for peatland development and persistence—to investigate the future peatland distribution in the TP. SSP1–2.6 represents a low-emissions pathway with strong climate mitigation policies, where global net CO_2_ emissions become negative after 2075 AD. For the areas currently covered by the Zoige peatland complex, the CMIP6 model ensemble under SSP1–2.6 predicts a change in MAT of − 0.4 to + 4.3 °C for the 2100 AD compared to the modern baseline period (CR, 1961–1990). The MI increases by 2.5–2.7 for the same time period and scenario (see Supplementary Table 1 and Supplementary Fig. 2 online). Under SSP1–2.6, our simulations suggest that the suitable climate envelope for the Zoige peatland complex will shrink in 2100 AD and the peatland climate space will move westwards towards the central TP region mainly due to increases in precipitation and permafrost thawing in those regions (Fig. 3 and Supplementary Fig. S3 online). The late 21st-century cooling following the mid-century temperature peak under SSP1–2.6 will not be sufficient to re-establish suitable climatic conditions for peatland development, similar to findings on permafrost peatland distribution elsewhere^15^. Our results therefore suggest that large areas of the suitable climatic space seen in the baseline period may be lost by 2100 AD (Fig. 5 and Supplementary Fig. S4 online). TP peatlands over the coming decades may therefore provide important early indications of likely ecosystem trajectories elsewhere across the Arctic regions. Our simulations using the more pessimistic SSP5–8.5 (a higher emission scenario) indicate that the current Zoige peatland complex will be completely outside their climate envelope by 2100 AD (Fig. 5 and Supplementary Fig. S3 online). Instead, their optimum environmental and climate space will shift to the centre of the TP due to permafrost thaw, suggesting that new peatlands may develop in these areas (Fig. 5 and Supplementary Fig. S4 online).

## Discussion

### Environmental space of TP peatlands

Soil saturation is a requirement for peatland development, and, in fact, the coupling of soil moisture conditions and peatland vegetation emergence is well established^22^. Soil moisture is the balance of precipitation and evapotranspiration, which in turn is affected by the temperature and radiation. At the large regional scale soil moisture is primarily controlled by precipitation, slope, and aspect^23^. In our study, soil moisture index (MI) is one of the main variables controlling peatland distribution. For example, in the Zoige peatlands complex, mean annual precipitation is ~ 400 mm, which is relatively low, but high cloud coverage and low temperatures both reduce evapotranspiration resulting in a high MI (1.8–2.0). As a result, the Zoige peatlands complex is the largest and best developed in the TP. Additionally, the TP is a mountain region with rugged topography and steep slopes, which also constrain peatland expansion^24^. The uniquely extensive Zoige peatland complex in the TP has a relatively flat topography compared with the steeper regions in the rest of the TP, and our results show that slope is a key variable for the TP peatlands distribution, as flatter ground (< 5°) tends to favour peatland growth^10,25^. Faster expansion rates in the Zoige peatlands complex region have also been associated with gentler slopes^25^. In the Zoige region, peatlands have also been found to develop largely in areas where the slope is less than 0.4°^25^). This is the case also in Finland, where gentle slopes of < 0.4° contribute to rapid peatland expansion^26^. Loisel and Yu^27^ also found the existence of a ‘slope threshold’ (< 0.5°) below which peat can rapidly expand laterally. However, differently to these gently sloping peatlands, some of the TP peatlands seem to exist on mid-slope section (above 5°) of the hillsides (e.g. Chadan peatland, see Supplementary Fig. S5 online), likely because the upper-slope section serves as catchment area that increases water supply to these areas (Y.F. Li et al., manuscript, under review).

Elevation may also have an influence on peatland development, altitude limits overall temperatures and also growing season lengths. In our investigation, there are no literature-based TP peatlands sites at an elevation greater than 5000 m. Peatlands in the TP according to our literature database are mostly concentrated at elevations between 3000 and 3500 m, but their total elevation range from 2500 to 5000 m is similar to other mountain peatlands in the North and South American continents^16^; see Supplementary Table S2 online). Although it has been established that mountain peatlands are generally sustained by precipitation and/or the perennial inflow of groundwater, likely stemming from a combination of rain and snowmelt (and glaciers in a few instances), it is uncertain how each factor contributes to overall productivity and if temperatures or growing season length play a significant role in plant productivity in this region^28^. Finally, the active layer thickness in permafrost-affected regions may be another important factor in peatland distribution in the TP, as peatlands tend to develop in the area of permafrost (active layer thickness < 2.3 m). This could be linked to the effect of permafrost as a water impermeable layer, which prevents water infiltration and allows surface water ponding. Overall, a complex combination of both climate and topographic factors ultimately determines the peatland distribution in the TP.

### Future environmental and climate space on the TP and changes to peatland distribution

Warming is expected to be more rapid in mountain regions^29^, and TP peatlands are expected to be highly sensitive to these rapidly changing climate conditions^30^. By 2100 AD, our simulations indicate that the region of optimum climate of peatland development may change greatly in the TP. Our results indicate that widespread losses of suitable environmental and climate space will occur even under a low warming scenario (SSP1–2.6) in the Zoige peatland complex. The peatlands climate envelope will move northwards in the low emission scenario and towards the west in the high emissions scenario due to permafrost degradation. There are caveats to these findings, as our model does not account for peatland persistence through self-regulating processes^31^ and the spatial heterogeneity of vegetation stability under a changing climate^32^, or the impacts of the CO_2_ fertilisation accompanying climate changes. Also, the time-lag of the response of vegetation to climate^32^ and human activities increase the uncertain of the results. Although peat accumulation in mountain peatlands in the TP may be controlled by temperature and other factors, soil moisture is likely to be the main control on peatland extent^23^. Despite the weaker relationship with temperature and the complexity associated with hydrological processes and water supply^33^, projected warming could result in more significant decreases in soil moisture and increases in evapotranspiration, which can have significant effects on water table variability peatland plant community composition, and ecosystem structure, and in turn productivity^34^. Also, predictions of precipitation are highly uncertain^35–37^, which will be key to the future fate of the TP peatlands. Still, this study highlights the potential threats to existing peatlands in the Zoige peatland complex.

In summary, the modern distribution of TP peatlands is well captured by a bioclimatic model (PeatStash) driven by MI, slope, minimum mean annual temperature, maximum mean annual temperature, and active layer thickness, especially for the Zoige peatland complex region (without active layer thickness, as the region is not affected by permafrost), where model output statistics are generally good. The future peatland distribution of peatlands across the Zoige peatland complex region is predicted to shrink by 2100 AD even under a low emissions scenario (SSP1–2.6), while the peatland suitable areas will shift westwards over the TP, especially under a high emissions scenario (SSP5–8.5). Model performance for the whole of the TP is hindered by poor climate data and available peatland maps. Having high quality ground truthing data, including peatland vegetation diversity, is key to understanding the present and future peatland distribution. Mapping of peatlands in the TP remains a priority if we are to understand carbon stocks and impacts of climate on these sensitive ecosystems.

### Limitations and future research

Various types of peatlands on the TP likely have different environmental space and thresholds (see Supplementary Fig. S6 online). We have focused on one such peatland type: the Zoige peatland complex in the northeastern TP dominated by *Carex muliensis* and *Kobresia tibetica*. We have also observed other types of peatlands in the field (see Supplementary Fig. S5 online). Peatlands in the central TP near the source region of the Yangtze River are distributed on mid-hillslope and are dominated by *Kobresia* spp. The region has an arid and cold climate at an elevation of 4800 m asl. Another peatland type in the Hengduan Mountains at the SE edge of the TP at subtropical latitude is distributed in valley bottoms dominated by *Carex*, *Sphagnum*, and some low-growing bamboos, under a relatively mild and wet climate. The large range of environments in the TP increases complexity in any modelling exercise trying to capture all these peatland types with one set of thresholds. If better maps existed for these different peatland types, it would be possible to develop climate thresholds for each of them and, in this way, improve our understanding of their drivers and likely future fates.

Model performance is highly reliant on the quality of the input data, in particular the peat map and environmental and topographic data used. The accuracy of this input data is key to the setting up of thresholds and model performance^38^. For example, PeatStash predicts the distribution of blanket bogs in Great Britain with reasonably high accuracy^13^ and also the Zoige peatland complex, where accurate mapping exists. On the other hand, model outputs for the whole TP are characterised by low Kappa statistics. The lack of accurate high-resolution data on both environmental/topographic variables and peatland distribution precluded comparisons with model outputs. PEATMAP was developed as a wetland map using survey data from various sources and remote sensing^20^, but for the TP region, PEATMAP is likely to overestimate peatland extent in some regions^39^ (see Supplementary Fig. S7 online). For example, in the Qaidam Basin, a hydrologically-closed arid intermountain basin, the MI is less than 0.3 and precipitation is consistently low (< 70 mm/year), suggesting an environment devoid of peatlands (see Supplementary Fig. S7 online). We checked Google Earth images of this region, and indeed, no peatlands appear to be present in the region even though they are predicted as such by PEATMAP. False positives given by PEATMAP are usually sites that are too dry or too steep and where peatlands cannot possibly develop^39^. Additionally, PEATMAP fails to include many of our literature-based TP peatlands study sites, that is, the map underestimates peatland extent in other areas (see Supplementary Fig. S8 online). Additionally, another peat map derived from machine learning analysis of large data sets of environmental variables^40^ (see Supplementary Fig. S9 online) and the European Space Agency (ESA) herb wetland map (https://viewer.esa-worldcover.org/) (see Supplementary Fig. S10 online) have similar problems in the TP, most likely due to the complexity of the TP topography and associated peatland systems. Therefore, improved peatland mapping in the TP should be a research priority.

Other sub-grid factors may be important for mountain peatlands, such as local topography and microclimate, but are not captured in our modelling exercise^13^. The DEM data available at 30-m resolution is probably too coarse to resolve valley peatlands and any other small sized peatland (Table 3 and Supplementary Fig. S1 online). Finally, meteorological stations are concentrated in the eastern TP, and the number of weather stations is sparse elsewhere in the TP. CRU data were calculated using interpolation of station data^41^, so the number of stations used to derive the data likely impacts its reliability, which then impacts model performance in those regions. There are obvious differences between the CRU data and the local meteorological station data (Table 3). For example, the mean annual temperature is both overestimated or underestimated, especially in the western part of the TP. There are only a few meteorological stations (see Supplementary Fig. S11 online) in the region between 65°E and 90°E longitudes, so both the CRU climate data and CMIP6 products which are calculated using interpolation of the meteorological stations will suffer from a loss of accuracy^42,43^. Finally, our bioclimatic modelling may also be limited in the central and western TP (80°E–95°E), because it can only capture the precipitation instead of hydrologic process that may feed the peatlands, such as lakes or glacial outwash, which are widely distributed in this area but beyond the capabilities of PeatStash. Moreover, most peatlands in the region are closed to lakes (Fig. 1), which probably are the water source of peatlands rather than the precipitation.

**Table 3:** Difference between CRU data and local meteorological station data for different peatland sites or regions.

Peat sites/regions	Data Source	MAT ( °C )	PJJA (mm)	Slope (°)
Libilv peatland	CRU data	7.9	435.4	1.9
	Meteorological station	14.7	567.0	~ 0.0
Dangha peatland	CRU data	9.7	436.0	11.5
	Meteorological station	14.7	567.0	~ 0.0
Chadan peatland	CRU data	− 3.6	326.5	0.5
	Meteorological station	− 1.1	325.0	0.3
Zoige peatland complex	CRU data	2.3	354.0	1.0
	Meteorological station	1.1	~ 480.0	< 0.4
The western TP	CRU data	− 2.2	70.7	–
	meteorological station	1.8	1.9	–

*MAT* mean annual temperature, *PJJA* precipitation of June, July and August.

## Methods

We examined three overlapping environmental and climatic spaces covering the following areas (Fig. 2): (1) all TP peatlands as shown by PEATMAP, (2) the Zoige peatland complex, this is a well-studied peatland area where we have high confidence that peatlands are well mapped, and (3) the literature-based TP peatlands study sites. We compared the broader climate envelope occupied by all of the TP peatlands to that of the Zoige peatland complex (Fig. 2). We used the environmental space analysis to select the most significant variables. Our final model includes MI and slope as significant predictors, alongside the minimum MAT, maximum MAT, and active layer thickness, which maximised model informedness when predicting the modern TP peatlands distribution from modern environment data. We compared the environmental and climate space of TP peatlands in the PEATMAP and literature-based TP peatlands sites to the more general climate distribution of all ecosystems in the TP.

### Study regions

The Tibetan Plateau (TP, 70–105°E, 25–45°N) (Fig. 1) is the world’s highest and largest plateau, with an average elevation exceeding 4500 m with mountain ranges and large brackish lakes. The TP contains the headwaters of major rivers in the surrounding regions and serves as a “water tower”^44^. The climate in the TP is affected by the westerlies, the East Asian summer monsoon, and the South Asian summer monsoon. Mean annual precipitation ranges from 20 to 2000 mm, and the mean annual temperature varies from − 6 to 20 °C (https://www.tibetol.cn/html/2013/gy_0513/968.html). Permafrost occurs over extensive parts of the plateau. The 5086 km^2^ peatland region, almost half of the peatland area in China, stores approximately 1.49 Pg C, which accounts for 68% of the total peatland C storage in China^19^. We have divided the TP into four distinct regions for the MI analysis to summarize the different peatland development in different region (Figs. 1 and 2) (Zoige peatland complex: 102–103°E, 32–35°N; central region: 93–102°E, 32–35°N; western region: < 93°E; and southern region: > 93°E, < 32°N).

### Peatland map and peat sites

We use PEATMAP^20^ as the present-day peatland map for the TP peatlands. This map incorporates information derived from digitised soil maps, wetland databases and satellite imagery, including bogs, fens, swamps, and freshwater marshes. This map excludes lakes and river wetlands in the TP. For simplicity, we define the Zoige peatland complex in the longitude range of 102–103°E and the latitude range of 32–35°N, as captured also in PEATMAP.

To collate a database of Tibetan peatland locations, we searched the terms “Tibet peat/peatland”, “Tibetan plateau”, “peat/peatland”, “Xizang peat/peatland”, “Qinghai peat”, “Zoige/Ruoergai” and “Hongyuan” in Google Scholar and Baidu Scholar (using terms in both English and Chinese) based on the dataset from Liu et al.^45^ and Wang et al.^46^. A total of 309 literature-based TP peatland study sites were extracted (see Supplementary Table S3 online). More than 100 sites were from surveys before the 1990s (these site locations may be less accurate due to old hand-held GPS receiver). We therefore checked every peatland site on Google Maps, and removed those sites obviously located in lakes, forests, and residential areas. A total of 309 literature-based TP peatlands sites remained and were used for the peatland environmental analysis. It is likely that a number of additional peatlands may exist which have not been recorded in the literature, particularly in the remote regions that are understudied, but the effect of these missing data is impossible to quantify in the current study. We set the boundary of the TP following the newest edition of the National Tibetan Plateau/Third Pole Environment Data Center (TPDC, https://www.tpdc.ac.cn/en/)^47,48^.

### Climate and environment data

The modern climate data were extracted from CRU CL v. 2.0^41^ (https://crudata.uea.ac.uk/cru/data/hrg/tmc/), which is a gridded climatology of 1961–1990 monthly means with a spatial resolution 10′ × 10′ (longitude × latitude). The slope^49–53^ and active layer thickness (ALT)^54^ data are from the National Tibetan Plateau/Third Pole Environment Data Center (TPDC, https://www.tpdc.ac.cn/en/). Out of the four available future climate scenarios, we chose the lowest and highest warming scenarios (Shared Socioeconomic Pathway (SSP) 1–2.6 and SSP5–8.5) to represent the range of possible future climate from four CMIP6 models, which are FGOALS_g3, HadGEM3_MM, MIROC_ES2L, and GFDL_ESM4 (see Supplementary Fig. S12, Supplementary Fig. S13, and Supplementary Fig. S14 online)^55^ (https://esgf-node.llnl.gov/search/cmip6/; https://cds.climate.copernicus.eu). But due to the limitations of the ALT data source for CMIP6, we used earlier ALT results for Representative Concentration Pathway (RCP) 2.6 and RCP 8.5 to represent the low-to-high scenarios corresponding to the CMIP6 climate data^54^.

We conducted all of our analyses at a spatial resolution of 10′ latitude × 10′ longitude, to match the resolution of our modern and future environmental and climate datasets. A cumulative total of 10,658 grids were extracted from the CRU data using bilinear interpolation over the bicubic spline approach covering the PEATMAT, Zoige peatland complex and the literature-based TP peatlands sites of the TP. We resampled the PEATMAP data, the literature-based TP peatlands sites, future climate, slope, and elevation data to the same spatial resolution as the modern CRU climate data. All the climate and non-climate data used for environmental and climate space analysis were extracted (1) for each of the grids corresponding to the 309 literature-based TP peatlands sites (covering a total of 65 grids) and also (2) for the PEATMAP grid cells that have a minimum of 12.2% peat coverage (1305 grids), which is greater than the real coverage (< 2%)^18^. CMIP6 temperature values were converted from Kelvin to degrees Celsius, precipitation values were converted from mean precipitation flux (kg m^−2^ s^−1^) to mean monthly totals (mm), and the cloud cover percentage was converted to % sunshine. We then downscaled and bias-corrected the CMIP6 outputs to a 10′ × 10′ spatial resolution using bilinear interpolation over the bicubic spline approach, because this approach is more widely used and because another bicubic interpolation approach can cause unrealistically high climatic variability^56^. SSP1–2.6 and SSP5–8.5 represent a low emission and a very high emission pathway, resulting in the global radiative forcing by 2100 AD of 2.6 and 8.5 W m^−2^, respectively.

### PeatStash climate envelope model and its evaluation metrics

We used the PeatStash model to capture the present-day distribution of TP peatlands in the Zoige peatland complex. The PeatStash model, a bioclimate envelope model, has been used to predict the future/past blanket peatland distribution^13,57^. The model for blanket peatlands is based on mean annual temperature, mean temperature of the warmest, and moisture index (MI). MI is calculated from the mean monthly temperature, precipitation, and fractional sunshine hours^9^:

$${\text{MI}} = {\text{P/PET,}}$$

where P is the mean annual precipitation (mm) and PET is the mean annual potential evapotranspiration (mm). We substitute an estimate of the equilibrium evapotranspiration instead of PET, which is a function of net radiation and temperature only. For empirical relationships among different moisture indices, including MI as calculated here, see Harrison et al.^58^.

In order to understand the main variables controlling peatland extent in the TP, we used the current climatic and environmental space to determine the main variables controlling peatland extent and their thresholds. We evaluated the predictive classifications output by the PeatStash model using three complementary evaluation metrics: accuracy, sensitivity and Kappa statistics. Accuracy evaluates the proportion of correctly classified cases (both presence and absence). Sensitivity was defined as the total number of recorded presences correctly predicted, as a fraction of the total number of presences recorded in the data^59^. Kappa statistics is a simply derived index that measures the proportion of all possible cases of presence or absence that are predicted correctly by a model after accounting for chance^60^.

### Supplementary Information

Supplementary Information.

## Data Availability

Data used in the study is available from the authors on a reasonable request.
