# Supplementary material for: Topographic and climatic controls of peatland distribution on the Tibetan Plateau
Source: Sci Rep. 2023 Sep 8;13:14811. doi: 10.1038/s41598-023-39699-x (PMC10491611; doi:10.1038/s41598-023-39699-x)
Supplement: Supplementary file 1 — Supplementary Information. [file 41598_2023_39699_MOESM1_ESM.docx]

**Supplementary**

Supplementary Table S1. Difference between the mean annual climate projected for 2100 AD and the modern baseline period (CR, 1961–1990) from different climate models under scenarios of low-to-high warming SSP (Shared Socioeconomic Pathway) 1-2.6 and SSP5-8.5.

| Model | scenario | ΔMAT (℃) | ΔPre (mm) | ΔSunshine (%) | ΔMI | Δ ALT (m) |
| --- | --- | --- | --- | --- | --- | --- |
| Basal (CRU) |  | -1.3 | 471.9 | 61.1 | 1.0 | 2.3 |
| MIROC-ES2L | SSP1-2.6 | 2.9 | 730.9 | -12.1 | 1.7 | -- |
|  | SSP5-8.5 | 8.2 | 836.0 | -11.9 | 1.6 | -- |
| FGOALS-g3 | SSP1-2.6 | -3.8 | 566.4 | -21.2 | 2.1 | -- |
|  | SSP5-8.5 | 0.4 | 829.7 | -23.7 | 2.3 | -- |
| HadGEM3-GC31-MM | SSP1-2.6 | 1.1 | 376.1 | -21.9 | 0.9 | -- |
|  | SSP5-8.5 | 6.7 | 469.0 | -19.7 | 0.8 | -- |
| GFDL-ESM4 | SSP1-2.6 | -1.7 | 525.4 | -25.4 | 1.8 | -- |
|  | SSP5-8.5 | 2.1 | 604.2 | -22.6 | 1.5 | -- |
| Mean value | SSP1-2.6 | -0.4 | 551.7 | -20.3 | 2.5 | 0.2 |
|  | SSP5-8.5 | 4.3 | 688.7 | -19.6 | 2.7 | 0.5 |

MAT is mean annual temperature

Pre is the annual precipitation

MI is the soil moisture index

ALT is active layer thickness

Because the mean ALT =$\Sigma ALT/area$. The ALT area of basal, low and high scenarios is 1.2x10^6^ km^2^, 9.7x10^5^km^2^, and 4.7x10^5^km^2^, respectively.That means the permafrost area is shrinking with global warming, while the mean ALT is increasing.

Supplementary Table S2. Comparison of different high-elevation peatland regions in the world.

| Region | Elevation (m) | MI | MAT (℃) | Pre (mm) | Slope (°) |
| --- | --- | --- | --- | --- | --- |
| Tibetan Plateau | 2600-5200 | 0-2.2 | -6-6 | 30-700 | 0-10 |
| Andes Mountain | 2500-4900 | -- | 6.8 | 500-1000 | -- |
| New Guinea | 2800-4200 | -- | <10 | 2500-4000 | -- |
| Rocky Mountain | 2700-3800 | -- | -- | 340-1700 | 0-5 |
| Changbai Mountains | 600-2700 | -- | 2-5 | 600-1000 | -- |

MI is soil moisture index

MAT is mean annual temperature

Pre is the annual precipitation

Supplementary Table S3. A total of 309 literature-based TP peatland study sites based on Liu et al. (2020) and Wang et al. (2014).

|  | Site name | Latitude (N) | Longitude (E) | References |
| --- | --- | --- | --- | --- |
| 1 | Baihe | 33.25 | 102.50 | Chai, 1990. |
| 2 | Longriba | 32.43 | 102.37 | Chen et al., 2014. |
| 3 | Hongyuan | 32.75 | 102.57 | Chen et al., 2014. |
| 4 | Riganqiao | 33.10 | 102.65 | Chen et al., 2014. |
| 5 | Chaha’erqiao | 33.53 | 102.85 | Chen et al., 2014. |
| 6 | DenahequS | 33.53 | 102.80 | Chen et al., 2014. |
| 7 | Naluoqiao | 33.63 | 102.63 | Chen et al., 2014. |
| 8 | HuahuS | 33.92 | 102.87 | Chen et al., 2014. |
| 9 | Laizipo | 31.00 | 101.10 | China Quaternary Research Committee, 1990. |
| 10 | Tuotuo | 31.20 | 100.70 | China Quaternary Research Committee, 1990. |
| 11 | Aba County | 33.17 | 101.68 | China Quaternary Research Committee, 1990. |
| 12 | Jiuzhi County | 33.42 | 101.82 | China Quaternary Research Committee, 1990. |
| 13 | Axi Town | 33.85 | 102.88 | China Quaternary Research Committee, 1990. |
| 14 | Tangke Town | 33.88 | 102.92 | China Quaternary Research Committee, 1990. |
| 15 | Gahai | 34.15 | 102.30 | China Quaternary Research Committee, 1990. |
| 16 | Haogeleng | 34.70 | 101.53 | China Quaternary Research Committee, 1990. |
| 17 | Meiren Town | 34.97 | 103.27 | China Quaternary Research Committee, 1990. |
| 18 | EBoTA | 32.53 | 100.93 | Du et al., 2021. |
| 19 | Riganqiao | 33.10 | 102.63 | Guo et al., 2012. |
| 20 | Tangke | 33.45 | 102.63 | Guo et al., 2012. |
| 21 | NAQ18 | 32.53 | 91.47 | Kaiser et al., 2008. |
| 22 | Targo | 32.53 | 86.67 | Karoline,2013. |
| 23 | HYLK1 | 32.53 | 102.52 | Large et al.,2009. |
| 24 | Hong Yuan | 33.50 | 102.50 | Large et al.,2009. |
| 25 | Hongyuan | 32.53 | 102.52 | Li et al., 2012 |
| 26 | KE | 32.53 | 101.10 | Liu et al., 2013 |
| 27 | GLC | 32.53 | 95.60 | Liu et al., 2022 |
| 28 | ZBC | 32.53 | 92.38 | Liu et al., 2022 |
| 29 | Qinonggou | 32.53 | 90.97 | Meng et al., 2018 |
| 30 | Yuexi | 32.53 | 102.95 | Peng et al., 2021. |
| 31 | Kekehe East | 33.12 | 101.02 | Schlütz et al., 2009. |
| 32 | Kekehe West | 33.15 | 101.00 | Schlütz et al., 2009. |
| 33 | Magenacuo | 33.20 | 101.20 | Schlütz et al., 2009. |
| 34 | Ekuo River | 33.22 | 100.97 | Schlütz et al., 2009. |
| 35 | EkuoRiver | 33.22 | 100.97 | Schlütz et al., 2009. |
| 36 | Jiea Basin | 33.27 | 101.40 | Schlütz et al., 2009. |
| 37 | Ha′acuo Valley | 33.30 | 101.22 | Schlütz et al., 2009. |
| 38 | Ximenco Valley | 33.32 | 101.12 | Schlütz et al., 2009. |
| 39 | Jiukehe | 33.35 | 101.02 | Schlütz et al., 2009. |
| 40 | Ximenco Valley | 33.35 | 101.10 | Schlütz et al., 2009. |
| 41 | Lerzha River | 33.35 | 101.02 | Schlütz et al., 2009. |
| 42 | Jiukehe | 33.37 | 101.02 | Schlütz et al., 2009. |
| 43 | Jiukehe River | 33.37 | 101.02 | Schlütz et al., 2009. |
| 44 | Ximenco Valley | 33.40 | 101.10 | Schlütz et al., 2009. |
| 45 | Ximenco Valley | 33.42 | 101.10 | Schlütz et al., 2009. |
| 46 | Nigequ Valley | 33.42 | 101.20 | Schlütz et al., 2009. |
| 47 | Zhongdiannapaihai | 27.82 | 99.70 | Sun et al.,1998. |
| 48 | Daocheng | 29.03 | 100.30 | Sun et al.,1998. |
| 49 | Daofu | 30.98 | 101.12 | Sun et al.,1998. |
| 50 | Longri | 32.12 | 102.77 | Sun et al.,1998. |
| 51 | Zhuqing Basin | 32.13 | 98.87 | Sun et al.,1998. |
| 52 | Erigrnma | 32.13 | 100.35 | Sun et al.,1998. |
| 53 | Longriba | 32.45 | 102.71 | Sun et al.,1998. |
| 54 | Eduoma | 32.53 | 99.38 | Sun et al.,1998. |
| 55 | Sizhai | 32.53 | 102.25 | Sun et al.,1998. |
| 56 | Seyaqu1 | 32.53 | 102.27 | Sun et al.,1998. |
| 57 | Anqumuqiao | 32.53 | 103.43 | Sun et al.,1998. |
| 58 | Xinluhai | 32.53 | 99.12 | Sun et al.,1998. |
| 59 | Sandaoqiao | 32.53 | 103.28 | Sun et al.,1998. |
| 60 | Molashan | 32.53 | 99.53 | Sun et al.,1998. |
| 61 | Rangdongkegu | 32.53 | 100.33 | Sun et al.,1998. |
| 62 | Xiewakegu | 32.53 | 100.18 | Sun et al.,1998. |
| 63 | Youyicountry | 32.53 | 99.83 | Sun et al.,1998. |
| 64 | Tarugu | 32.53 | 100.32 | Sun et al.,1998. |
| 65 | Herie | 32.53 | 98.95 | Sun et al.,1998. |
| 66 | Longrixiang | 32.53 | 100.67 | Sun et al.,1998. |
| 67 | Ganzi | 32.53 | 101.98 | Sun et al.,1998. |
| 68 | Maoyaba | 32.53 | 99.68 | Sun et al.,1998. |
| 69 | Jiawa | 32.53 | 100.60 | Sun et al.,1998. |
| 70 | Laolinkou | 32.53 | 103.13 | Sun et al.,1998. |
| 71 | Tari | 32.53 | 101.07 | Sun et al.,1998. |
| 72 | Xingyicuo | 32.53 | 100.07 | Sun et al.,1998. |
| 73 | Xiazha | 32.53 | 99.35 | Sun et al.,1998. |
| 74 | Daocheng | 32.53 | 100.25 | Sun et al.,1998. |
| 75 | Shiping-Baoxiu | 32.53 | 102.50 | Sun et al.,1998. |
| 76 | Seyaqu1 | 32.63 | 102.35 | Sun et al.,1998. |
| 77 | Hongyuan | 32.67 | 102.62 | Sun et al.,1998. |
| 78 | Ganeryinqiao | 32.70 | 98.68 | Sun et al.,1998. |
| 79 | HongYuan | 32.78 | 102.53 | Sun et al.,1998. |
| 80 | Dazhang | 32.78 | 100.07 | Sun et al.,1998. |
| 81 | Hongyuan | 32.78 | 102.55 | Sun et al.,1998. |
| 82 | Hongyuangong | 32.80 | 102.55 | Sun et al.,1998. |
| 83 | Jiaqu2 | 32.88 | 102.27 | Sun et al.,1998. |
| 84 | Caqie | 32.90 | 102.72 | Sun et al.,1998. |
| 85 | Hongyuan Mine II | 32.92 | 102.58 | Sun et al.,1998. |
| 86 | Amukexiang2 | 32.92 | 102.63 | Sun et al.,1998. |
| 87 | Xiangyong | 32.92 | 98.07 | Sun et al.,1998. |
| 88 | Shenmekuo | 32.92 | 102.62 | Sun et al.,1998. |
| 89 | Wengqu | 32.95 | 98.07 | Sun et al.,1998. |
| 90 | Aba2 | 32.98 | 101.60 | Sun et al.,1998. |
| 91 | Zhagerima | 33.07 | 98.07 | Sun et al.,1998. |
| 92 | Jianuo | 33.07 | 102.25 | Sun et al.,1998. |
| 93 | Congta | 33.10 | 99.65 | Sun et al.,1998. |
| 94 | Haqu1 | 33.12 | 102.95 | Sun et al.,1998. |
| 95 | Waqie | 33.12 | 102.63 | Sun et al.,1998. |
| 96 | Haizikou Lake | 33.12 | 102.63 | Sun et al.,1998. |
| 97 | Maiqu | 33.12 | 103.05 | Sun et al.,1998. |
| 98 | Sangrikuhe | 33.13 | 102.40 | Sun et al.,1998. |
| 99 | Riganqiao | 33.15 | 102.77 | Sun et al.,1998. |
| 100 | Riganqiao | 33.15 | 102.77 | Sun et al.,1998. |
| 101 | Riganqia o84--12 | 33.20 | 102.67 | Sun et al.,1998. |
| 102 | JiangcuoLake | 33.23 | 99.37 | Sun et al.,1998. |
| 103 | Haqu2 | 33.23 | 103.02 | Sun et al.,1998. |
| 104 | Cina- Qielang | 33.25 | 98.12 | Sun et al.,1998. |
| 105 | Baihe | 33.25 | 102.50 | Sun et al.,1998. |
| 106 | Niangdiquwo | 33.28 | 102.58 | Sun et al.,1998. |
| 107 | Zegerie | 33.28 | 101.55 | Sun et al.,1998. |
| 108 | Riganqiao84--11 | 33.33 | 102.53 | Sun et al.,1998. |
| 109 | Handu | 33.35 | 102.53 | Sun et al.,1998. |
| 110 | Tangke | 33.40 | 102.47 | Sun et al.,1998. |
| 111 | Qiujiema | 33.45 | 101.85 | Sun et al.,1998. |
| 112 | Naaiqu | 33.45 | 102.35 | Sun et al.,1998. |
| 113 | Banyou | 33.50 | 103.02 | Sun et al.,1998. |
| 114 | Tangdiqu | 33.53 | 102.43 | Sun et al.,1998. |
| 115 | Ouminikequ | 33.53 | 103.15 | Sun et al.,1998. |
| 116 | Eluo | 33.53 | 102.50 | Sun et al.,1998. |
| 117 | Denahe1 | 33.55 | 102.80 | Sun et al.,1998. |
| 118 | Kahaerqiao | 33.57 | 102.95 | Sun et al.,1998. |
| 119 | Kahaerqiao | 33.57 | 102.95 | Sun et al.,1998. |
| 120 | Requ | 33.58 | 103.10 | Sun et al.,1998. |
| 121 | Langquqiaoergan-Yeermoq | 33.60 | 102.10 | Sun et al.,1998. |
| 122 | Wanyantang | 33.62 | 102.22 | Sun et al.,1998. |
| 123 | Cuokuoer | 33.62 | 102.92 | Sun et al.,1998. |
| 124 | Naluoqiao | 33.62 | 102.77 | Sun et al.,1998. |
| 125 | Heiqingqiao | 33.65 | 102.62 | Sun et al.,1998. |
| 126 | Dehetang | 33.67 | 102.18 | Sun et al.,1998. |
| 127 | Hahemuqiao | 33.75 | 102.93 | Sun et al.,1998. |
| 128 | Luocha | 33.78 | 103.03 | Sun et al.,1998. |
| 129 | Nongyingqiaogan | 33.82 | 102.55 | Sun et al.,1998. |
| 130 | Xingcuo lake | 33.87 | 102.37 | Sun et al.,1998. |
| 131 | Reer | 33.88 | 102.97 | Sun et al.,1998. |
| 132 | Cheluoqu | 33.88 | 102.97 | Sun et al.,1998. |
| 133 | Heihemu chang | 33.92 | 102.57 | Sun et al.,1998. |
| 134 | Reerdaba | 33.95 | 102.85 | Sun et al.,1998. |
| 135 | Gunqiao | 33.95 | 102.10 | Sun et al.,1998. |
| 136 | Reerdaba | 33.95 | 102.85 | Sun et al.,1998. |
| 137 | Gaerquqiao | 33.97 | 102.45 | Sun et al.,1998. |
| 138 | Maqu | 34.00 | 102.07 | Sun et al.,1998. |
| 139 | Maqu | 34.00 | 102.07 | Sun et al.,1998. |
| 140 | Puwu | 34.07 | 103.63 | Sun et al.,1998. |
| 141 | Haqu | 34.28 | 102.95 | Sun et al.,1998. |
| 142 | Henan County | 34.70 | 101.53 | Sun et al.,1998. |
| 143 | Geke | 33.25 | 102.92 | Sun et al.,1998. |
| 144 | Ningqingqiao | 33.62 | 102.25 | Sun et al.,1998. |
| 145 | Adangqiao | 33.92 | 102.57 | Sun et al.,1998. |
| 146 | Longriba | 32.52 | 102.35 | Sun, 1983. |
| 147 | Hong Yuan II K | 32.80 | 102.55 | Sun, 1983. |
| 148 | Riganqiao | 33.10 | 102.63 | Sun,1992. |
| 149 | Kahareqiao | 33.33 | 102.58 | Sun,1992. |
| 150 | Maixigou | 33.67 | 102.38 | Sun,1992. |
| 151 | Zoige6 | 33.57 | 102.93 | The Ministry of Geology and Mineral Resources.,1986. |
| 152 | Zoige4 | 33.62 | 102.93 | The Ministry of Geology and Mineral Resources.,1986. |
| 153 | Zoige5 | 33.65 | 102.95 | The Ministry of Geology and Mineral Resources.,1986. |
| 154 | Hongyuan6 | 32.78 | 102.48 | Thelaus, 1992. |
| 155 | LBL | 27.62 | 98.59 | unpublished |
| 156 | DH | 27.78 | 98.47 | unpublished |
| 157 | CD21-C4 | 32.96 | 94.25 | unpublished |
| 158 | CD21-C1 | 32.96 | 94.25 | unpublished |
| 159 | CD21-C6 | 32.96 | 94.25 | unpublished |
| 160 | Hong Yuan II K | 32.80 | 102.55 | Wang et al., 1993. |
| 161 | Hongyuan3 | 32.78 | 102.52 | Wang et al., 2006. |
| 162 | WLR | 32.53 | 97.33 | Wang et al., 2018. |
| 163 | MDX | 32.53 | 96.25 | Wang et al., 2018. |
| 164 | Hong Yuan | 32.77 | 102.50 | Wang et al.,2003. |
| 165 | Zhongxun | 27.87 | 99.70 | Wang, 1984. |
| 166 | Daocheng County | 29.50 | 100.03 | Wang, 1984. |
| 167 | Hongyuan8 | 32.92 | 102.58 | Wang, 1984. |
| 168 | Jianuo Town | 33.03 | 102.22 | Wang, 1984. |
| 169 | Darigan | 33.28 | 101.25 | Wang, 1984. |
| 170 | Jiuzhi County | 33.32 | 101.25 | Wang, 1984. |
| 171 | Wuqilidigu | 33.42 | 102.83 | Wang, 1984. |
| 172 | Hongyuan4 | 32.77 | 102.50 | Wang, 2004. |
| 173 | Zhongxun | 32.53 | 99.70 | Wang, R.S., 1984. |
| 174 | BT-285 | 27.70 | 102.85 | Peng et al., 2021. |
| 175 | LJ | 32.53 | 101.33 | Xu et al., 2013. |
| 176 | Ebo | 32.53 | 1012.03 | Xu et al., 2013. |
| 177 | Waqie | 33.12 | 102.68 | Yin et al., 1991. |
| 178 | Waqie | 33.12 | 102.68 | Yin et al., 1991. |
| 179 | Luqu | 34.15 | 102.30 | Yin et al., 1991. |
| 180 | Zaerjiabugai | 34.15 | 102.30 | Yin et al., 1991. |
| 181 | Gawuyuewa | 34.90 | 98.20 | Yin et al., 1991. |
| 182 | Hongyaun2 | 32.78 | 102.52 | Yu et al., 2010. |
| 183 | HY2A | 32.53 | 102.87 | Yu et al., 2011. |
| 184 | HY2B | 32.53 | 102.87 | Yu et al., 2011. |
| 185 | hong yuan | 32.53 | 102.52 | Yuet al., 2006 |
| 186 | Longriba | 32.53 | 102.37 | Zhang, 2008. |
| 187 | jiangcuo Lake | 33.15 | 100.37 | Zhang, 2008. |
| 188 | Riganqia o84--7 | 33.25 | 102.58 | Zhang, 2008. |
| 189 | Wuqili | 33.32 | 102.82 | Zhang, 2008. |
| 190 | Wuqili | 33.33 | 102.85 | Zhang, 2008. |
| 191 | Jiangcuo Lake | 33.77 | 99.00 | Zhang, 2008. |
| 192 | ZB10-BD41 | 33.44 | 102.62 | Zhao et al 2014b. |
| 193 | Hongyuan | 33.45 | 102.62 | Zhao et al., 2011. |
| 194 | ZB10-BD9 | 32.71 | 102.52 | Zhao et al., 2014a. |
| 195 | ZB10-C7 | 32.71 | 102.38 | Zhao et al., 2014a. |
| 196 | ZB10-BD11 | 32.78 | 102.52 | Zhao et al., 2014a. |
| 197 | ZB10-C9 | 32.78 | 102.52 | Zhao et al., 2014a. |
| 198 | ZB10-BD6 | 33.04 | 103.03 | Zhao et al., 2014a. |
| 199 | ZB10-C6 | 33.07 | 103.03 | Zhao et al., 2014a. |
| 200 | ZB10-BD5 | 33.09 | 102.67 | Zhao et al., 2014a. |
| 201 | ZB10-BD36 | 33.10 | 102.67 | Zhao et al., 2014a. |
| 202 | ZB10-C14 | 33.10 | 102.67 | Zhao et al., 2014a. |
| 203 | ZB10-BD47 | 33.10 | 102.73 | Zhao et al., 2014a. |
| 204 | ZB10-BD34 | 33.11 | 102.63 | Zhao et al., 2014a. |
| 205 | ZB08-C1 | 33.45 | 102.63 | Zhao et al., 2014a. |
| 206 | ZB10-BD25 | 33.46 | 102.03 | Zhao et al., 2014a. |
| 207 | ZB10-C13 | 33.46 | 102.03 | Zhao et al., 2014a. |
| 208 | ZB10-BD22 | 33.46 | 102.03 | Zhao et al., 2014a. |
| 209 | ZB10-BD2B | 33.51 | 102.66 | Zhao et al., 2014a. |
| 210 | ZB10-C10 | 33.51 | 102.66 | Zhao et al., 2014a. |
| 211 | ZB10-BD26 | 33.62 | 102.95 | Zhao et al., 2014a. |
| 212 | ZB10-C15 | 33.67 | 102.50 | Zhao et al., 2014a. |
| 213 | ZB10-BD15 | 33.70 | 102.12 | Zhao et al., 2014a. |
| 214 | ZB10-BD7 | 33.71 | 102.38 | Zhao et al., 2014a. |
| 215 | ZB10-C12 | 33.72 | 102.12 | Zhao et al., 2014a. |
| 216 | ZB10-BD18 | 33.72 | 102.12 | Zhao et al., 2014a. |
| 217 | ZB10-BD20 | 33.72 | 102.12 | Zhao et al., 2014a. |
| 218 | ZB10-BD29 | 33.72 | 102.50 | Zhao et al., 2014a. |
| 219 | ZB10-BD31 | 33.72 | 102.50 | Zhao et al., 2014a. |
| 220 | ZB10-C3 | 33.78 | 102.84 | Zhao et al., 2014a. |
| 221 | ZB10-BD3 | 33.78 | 102.84 | Zhao et al., 2014a. |
| 222 | ZB08-C2 | 33.92 | 102.87 | Zhao et al., 2014a. |
| 223 | ZB10-BD27 | 33.93 | 102.87 | Zhao et al., 2014a. |
| 224 | CN11-C4 | 34.15 | 102.55 | Zhao et al., 2014a. |
| 225 | Zoige1 | 32.73 | 102.59 | Zhao et al., 2014b. |
| 226 | ZB10-BD12 | 32.78 | 102.52 | Zhao et al., 2014b. |
| 227 | ZB10-C8 | 32.78 | 102.52 | Zhao et al., 2014b. |
| 228 | ZB10-BD45 | 33.09 | 102.68 | Zhao et al., 2014b. |
| 229 | ZB10-BD4 | 33.09 | 102.67 | Zhao et al., 2014b. |
| 230 | ZB10-BD35 | 33.10 | 102.67 | Zhao et al., 2014b. |
| 231 | ZB10-BD46 | 33.10 | 102.70 | Zhao et al., 2014b. |
| 232 | ZB10-BD38 | 33.41 | 102.53 | Zhao et al., 2014b. |
| 233 | ZB10-BD39 | 33.41 | 102.53 | Zhao et al., 2014b. |
| 234 | ZB10-BD37 | 33.41 | 102.53 | Zhao et al., 2014b. |
| 235 | ZB10-BD40 | 33.45 | 102.62 | Zhao et al., 2014b. |
| 236 | ZB10-BD42 | 33.46 | 102.64 | Zhao et al., 2014b. |
| 237 | ZB10-BD24 | 33.46 | 102.03 | Zhao et al., 2014b. |
| 238 | ZB10-BD23 | 33.46 | 102.03 | Zhao et al., 2014b. |
| 239 | ZB10-BD1 | 33.47 | 102.64 | Zhao et al., 2014b. |
| 240 | ZB10-BD21 | 33.49 | 101.87 | Zhao et al., 2014b. |
| 241 | ZB10-BD2A | 33.51 | 102.66 | Zhao et al., 2014b. |
| 242 | ZB10-BD43 | 33.52 | 102.76 | Zhao et al., 2014b. |
| 243 | ZB10-BD48 | 33.52 | 102.76 | Zhao et al., 2014b. |
| 244 | ZB10-BD44 | 33.58 | 102.90 | Zhao et al., 2014b. |
| 245 | ZB10-BD16 | 33.70 | 102.12 | Zhao et al., 2014b. |
| 246 | ZB10-C11 | 33.71 | 102.12 | Zhao et al., 2014b. |
| 247 | ZB10-BD17 | 33.72 | 102.12 | Zhao et al., 2014b. |
| 248 | ZB10-BD19 | 33.72 | 102.11 | Zhao et al., 2014b. |
| 249 | ZB10-BD30 | 33.72 | 102.50 | Zhao et al., 2014b. |
| 250 | ZB10-BD28 | 33.72 | 102.49 | Zhao et al., 2014b. |
| 251 | Zoige3 | 33.75 | 102.50 | Zhao et al., 2014b. |
| 252 | ZB10-BD32 | 33.75 | 102.51 | Zhao et al., 2014b. |
| 253 | DN | 27.87 | 89.08 | Zhao, 1990. |
| 254 | ZGC | 28.48 | 91.30 | Zhao, 1990. |
| 255 | YZYC | 28.83 | 91.35 | Zhao, 1990. |
| 256 | LSH | 29.07 | 90.85 | Zhao, 1990. |
| 257 | DKZ | 29.30 | 89.12 | Zhao, 1990. |
| 258 | SS | 29.37 | 86.53 | Zhao, 1990. |
| 259 | NK | 29.45 | 84.50 | Zhao, 1990. |
| 260 | QD | 29.57 | 86.13 | Zhao, 1990. |
| 261 | CQ | 29.83 | 83.32 | Zhao, 1990. |
| 262 | NMC | 29.95 | 89.82 | Zhao, 1990. |
| 263 | MQH | 30.15 | 82.93 | Zhao, 1990. |
| 264 | SZZB | 30.18 | 88.47 | Zhao, 1990. |
| 265 | WMQ | 30.30 | 90.68 | Zhao, 1990. |
| 266 | LACMPYC | 30.50 | 81.22 | Zhao, 1990. |
| 267 | AZC | 30.83 | 82.72 | Zhao, 1990. |
| 268 | YZ | 30.85 | 89.32 | Zhao, 1990. |
| 269 | ZRNM | 30.85 | 85.15 | Zhao, 1990. |
| 270 | MDK | 30.88 | 92.40 | Zhao, 1990. |
| 271 | NQ | 31.08 | 91.12 | Zhao, 1990. |
| 272 | BG | 31.22 | 89.67 | Zhao, 1990. |
| 273 | AMZB | 31.27 | 82.67 | Zhao, 1990. |
| 274 | ZBBY | 31.30 | 84.05 | Zhao, 1990. |
| 275 | WRCZGC | 31.35 | 87.73 | Zhao, 1990. |
| 276 | SLC | 31.48 | 88.93 | Zhao, 1990. |
| 277 | CMCN | 31.62 | 83.57 | Zhao, 1990. |
| 278 | GPC | 31.82 | 83.03 | Zhao, 1990. |
| 279 | LGC | 31.83 | 84.28 | Zhao, 1990. |
| 280 | TGLSK | 31.97 | 91.45 | Zhao, 1990. |
| 281 | CMC | 31.97 | 83.43 | Zhao, 1990. |
| 282 | JRBC | 32.00 | 87.65 | Zhao, 1990. |
| 283 | DC | 32.08 | 84.38 | Zhao, 1990. |
| 284 | NEC | 32.22 | 82.05 | Zhao, 1990. |
| 285 | GZX | 32.27 | 83.67 | Zhao, 1990. |
| 286 | ZCCK | 32.45 | 81.97 | Zhao, 1990. |
| 287 | YBCK | 32.48 | 86.45 | Zhao, 1990. |
| 288 | Chawula | 32.53 | 93.33 | Zhao, 1990. |
| 289 | PDC | 32.67 | 87.48 | Zhao, 1990. |
| 290 | XXZBX | 32.95 | 82.38 | Zhao, 1990. |
| 291 | MC | 33.00 | 81.93 | Zhao, 1990. |
| 292 | BEC | 33.05 | 81.25 | Zhao, 1990. |
| 293 | LMX | 33.22 | 83.83 | Zhao, 1990. |
| 294 | JPX | 33.27 | 80.22 | Zhao, 1990. |
| 295 | BGC | 33.40 | 79.65 | Zhao, 1990. |
| 296 | MECX | 33.58 | 84.35 | Zhao, 1990. |
| 297 | XQ | 33.88 | 83.25 | Zhao, 1990. |
| 298 | MMC | 33.97 | 88.50 | Zhao, 1990. |
| 299 | JZCK | 33.97 | 80.53 | Zhao, 1990. |
| 300 | QTP | 32.53 | 102.87 | Zhen et al., 2021 |
| 301 | Hong Yuan | 32.80 | 102.52 | Zheng et al., 2011. |
| 302 | Hong Yuan | 32.80 | 102.52 | Zheng et al., 2011. |
| 303 | Zoige Basin | 32.80 | 102.52 | Zhou et al., 2002. |
| 304 | Hong Yuan | 32.77 | 102.52 | Zhou et al., 2010. |
| 305 | Zoige2 | 32.77 | 102.52 | Zhou et al., 2010. |
| 306 | Langmusi | 34.15 | 102.57 | Zhou et al., 2010. |
| 307 | Hongyuan | 32.53 | 102.50 | Zhou et al., 2011. |
| 308 | Xiaman | 33.77 | 33.77 | Zhou et al., 2016. |
| 309 | Huahu | 33.95 | 102.85 | Zhou et al., 2016. |


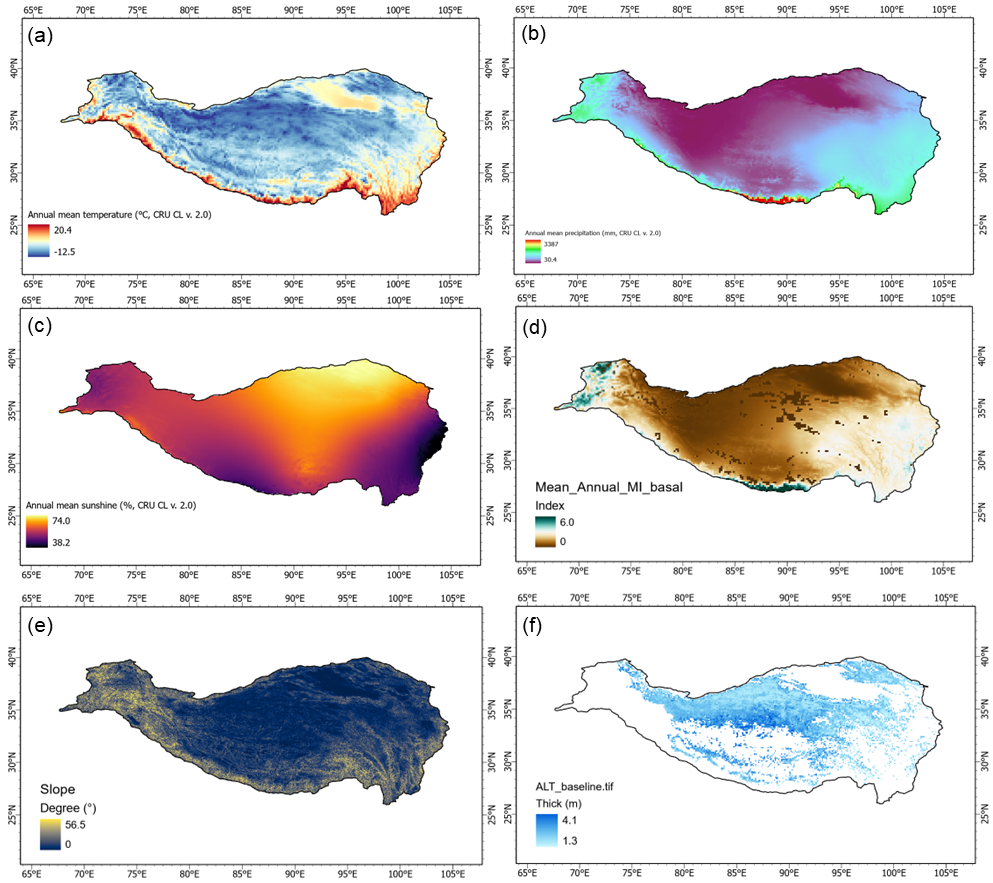


Supplementary Figure S1. The present-day annual mean climate data (CRU CL v. 2.0) for the TP, including (a) temperature, (b) precipitation, (c) sunshine, (d) soil moisture index (MI), (e) slope and (f) active layer thickness.


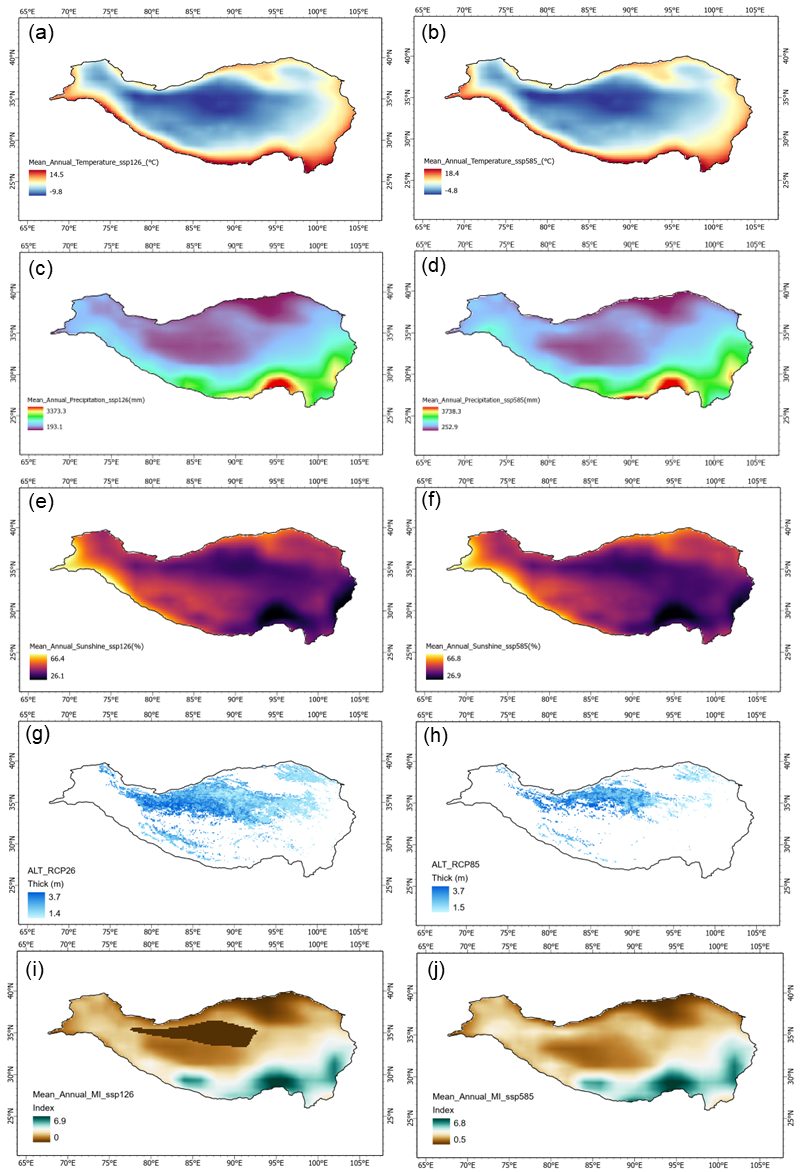


Supplementary Figure S2. Environmental (active layer depth) and climatic (MAT, MAP, sunshine, ALT, MI) data for the TP for the 2100 AD, showing four different model outputs (FGOALS_g3, HadGEM3_MM, MIROC_ES2L and GFDL_ESM4) under scenarios of low-to-high warming (Shared Socioeconomic Pathway (SSP) 1-2.6 (left) and SSP5-8.5(right)). (a) and (b) are MAT, (c) and (d) are MAP, (e) and (f) are sunshine, (g) and (h) are ALT, and (i) and (j) are MI.


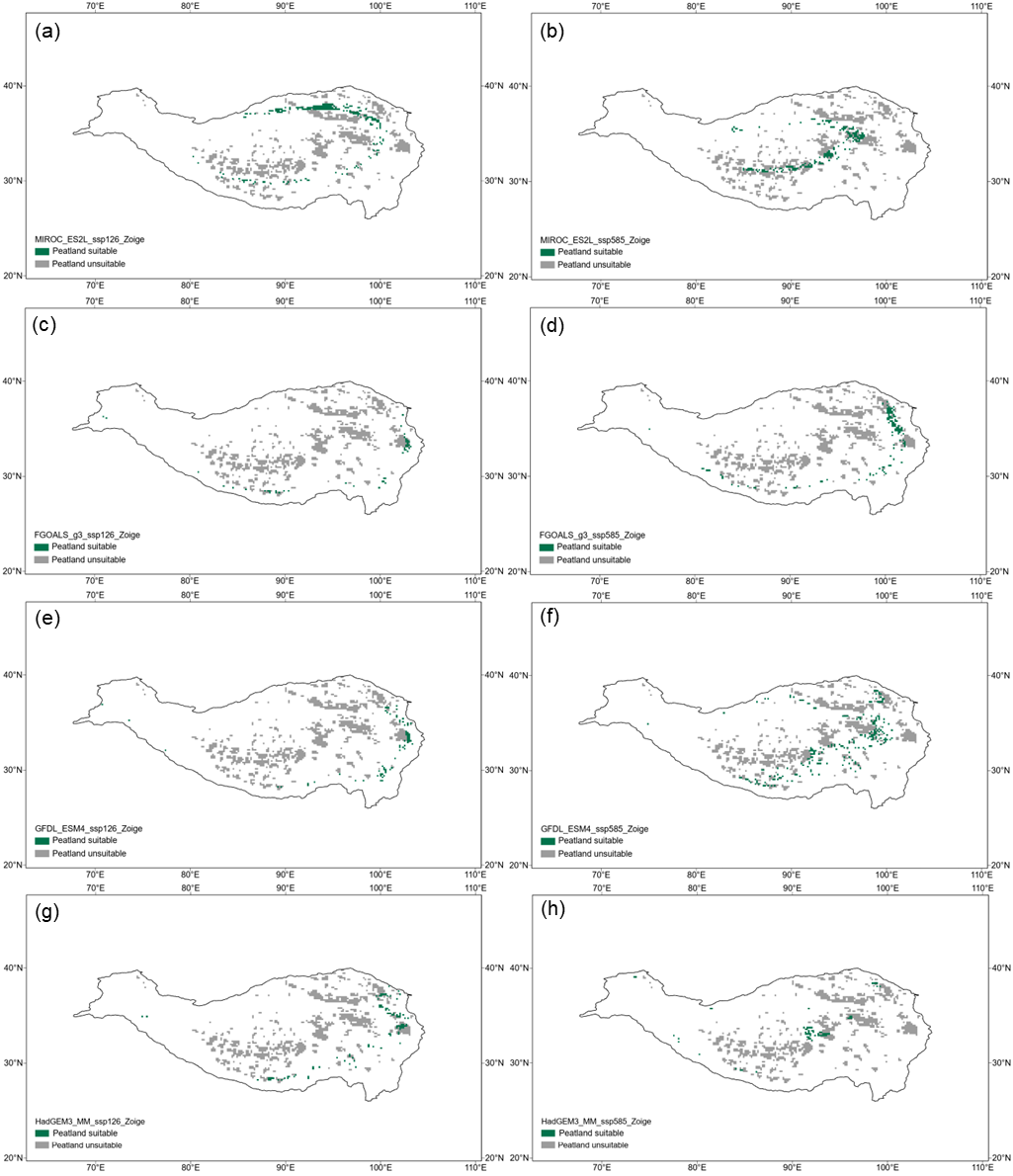


Supplementary Figure 3. Model predictions of peatland extent using different climate model outputs. (a) and (b), MIROC_ES2L model. (c) and (d), FGOALS_g3 model. (e) and (f), GFDL_ESM4 model. (g) and (h), HadGEM_MM model. Projections are based on the Zoige peatland complexnarrow threshold from the different CMIP6 climate model in 2100 AD. The climate runs chosen reflect the two scenarios of Shared Socioeconomic Pathway (SSP) described in the IPCC’s Sixth Assessment Report. Left, SSP1-2.6. Right, SSP5-8.5.


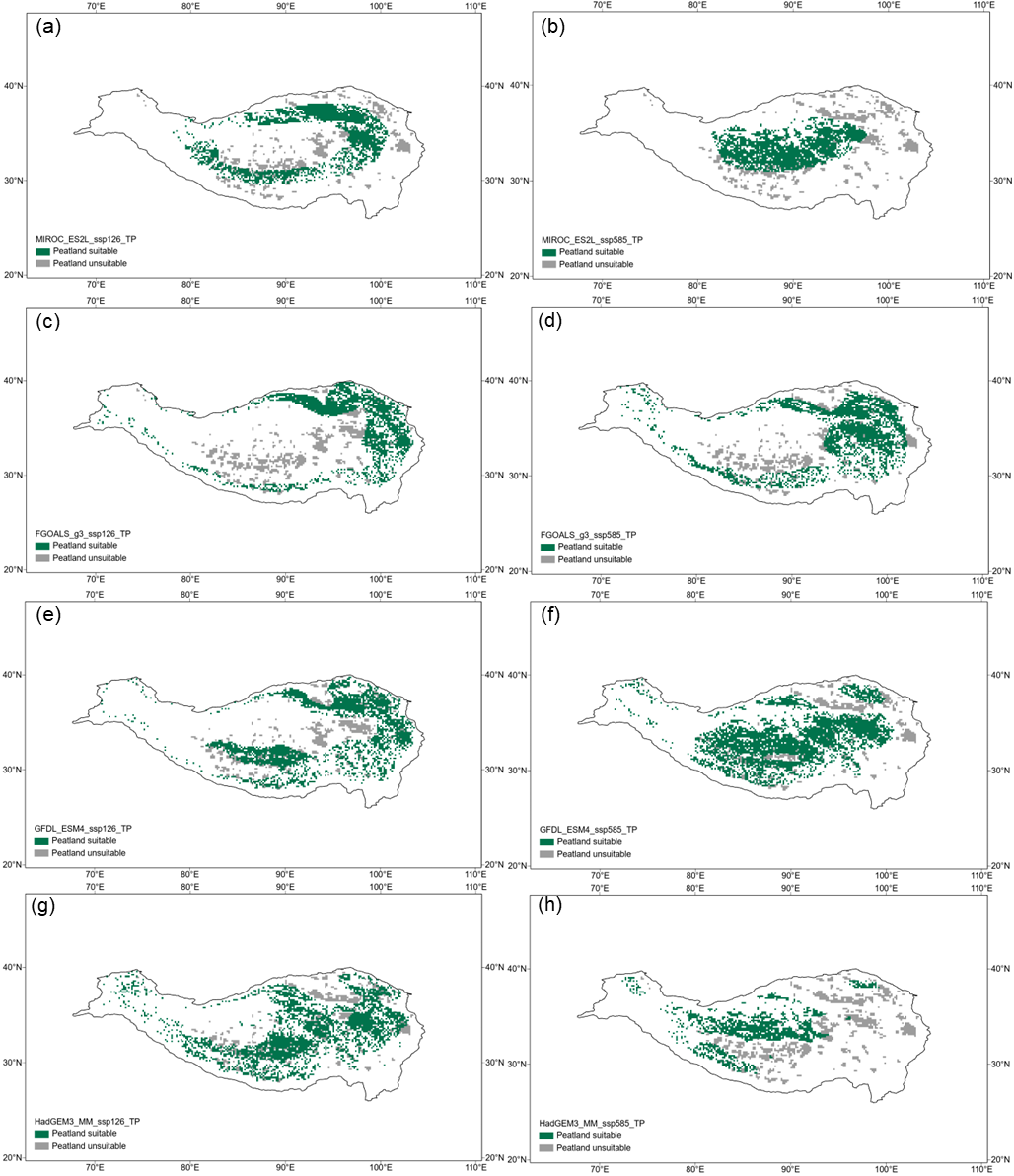


Supplementary Figure S4. Model predictions of peatland extent using different climate model outputs. (a) and (b), MIROC_ES2L model. (c) and (d), FGOALS_g3 model. (e) and (f), GFDL_ESM4 model. (g) and (h), HadGEM_MM model. Projections are based on the whole TPwide threshold from the different CMIP6 climate model in 2100 AD. The climate runs chosen reflect the two scenarios of Shared Socioeconomic Pathway (SSP) described in the IPCC’s Sixth Assessment Report. Left, SSP1-2.6. Right, SSP5-8.5.


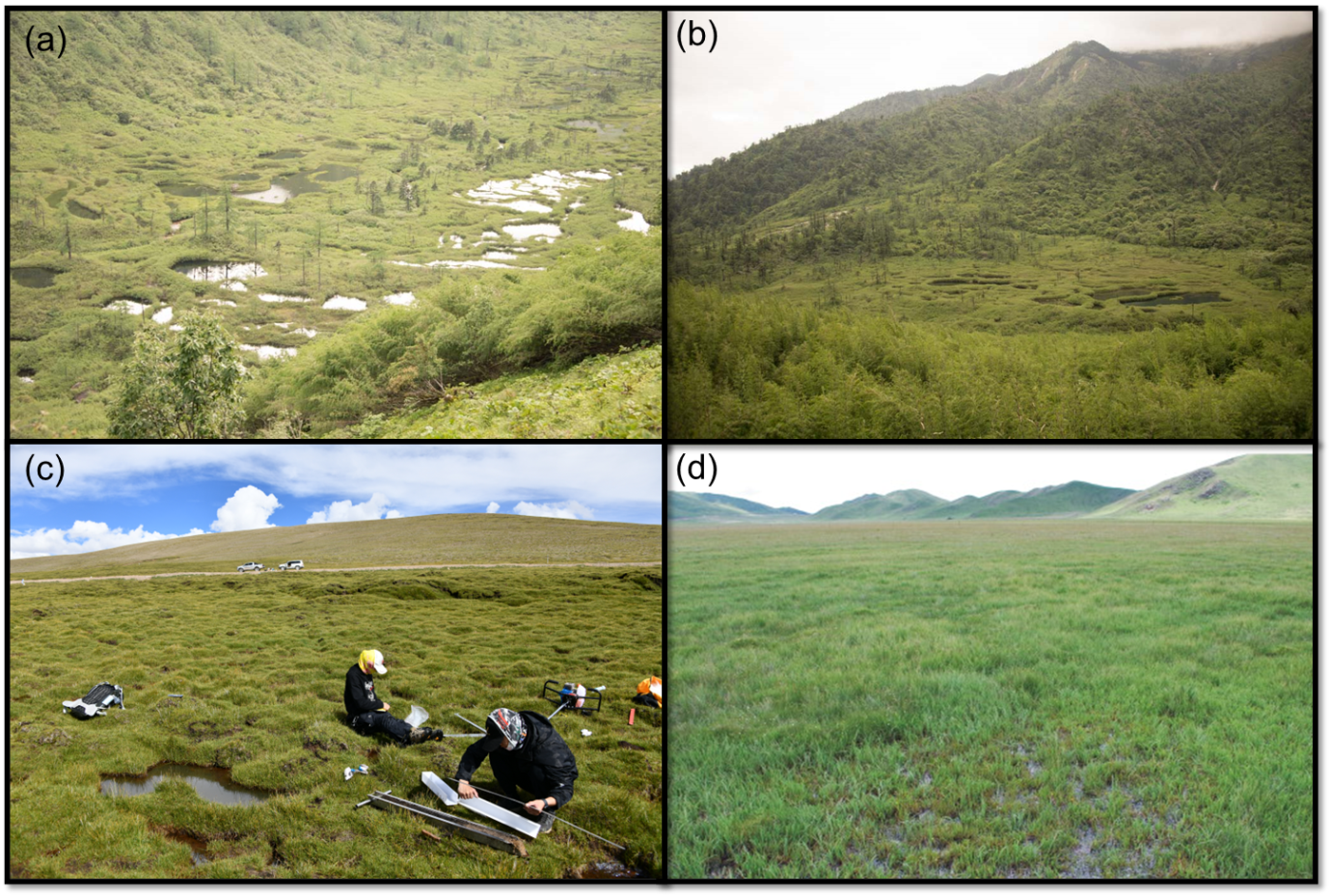


Supplementary Figure S5. Photographs depicting four different regions supporting different types of peatlands. (a) Libilv mountain peatland (LBL) and (b) Dangha peatland (DH) are located in the southeastern TP, where the dominant plants are *Sphagnum*, bamboo and *Carex*. (c) Chadan peatland (CD) is located in the central TP with the *Kobresia* being the dominant plants, and (d) Zoige peatland complex is in the northeastern TP, where *Carex* is the dominant plant species.


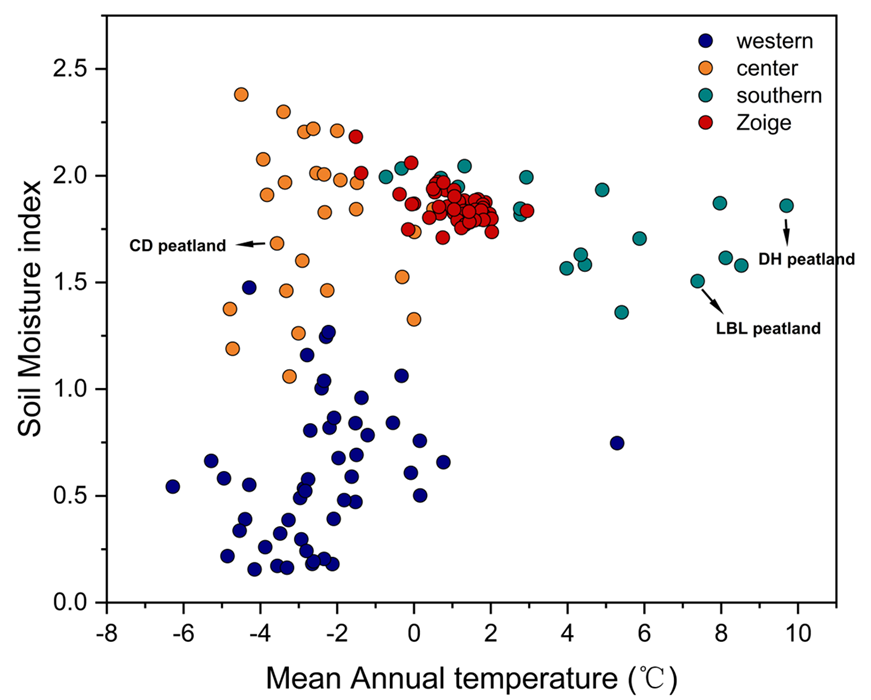


Supplementary Figure S6. Climate characterisation of different TP peatlands regions in a soil moisture index and the mean annual temperature space.


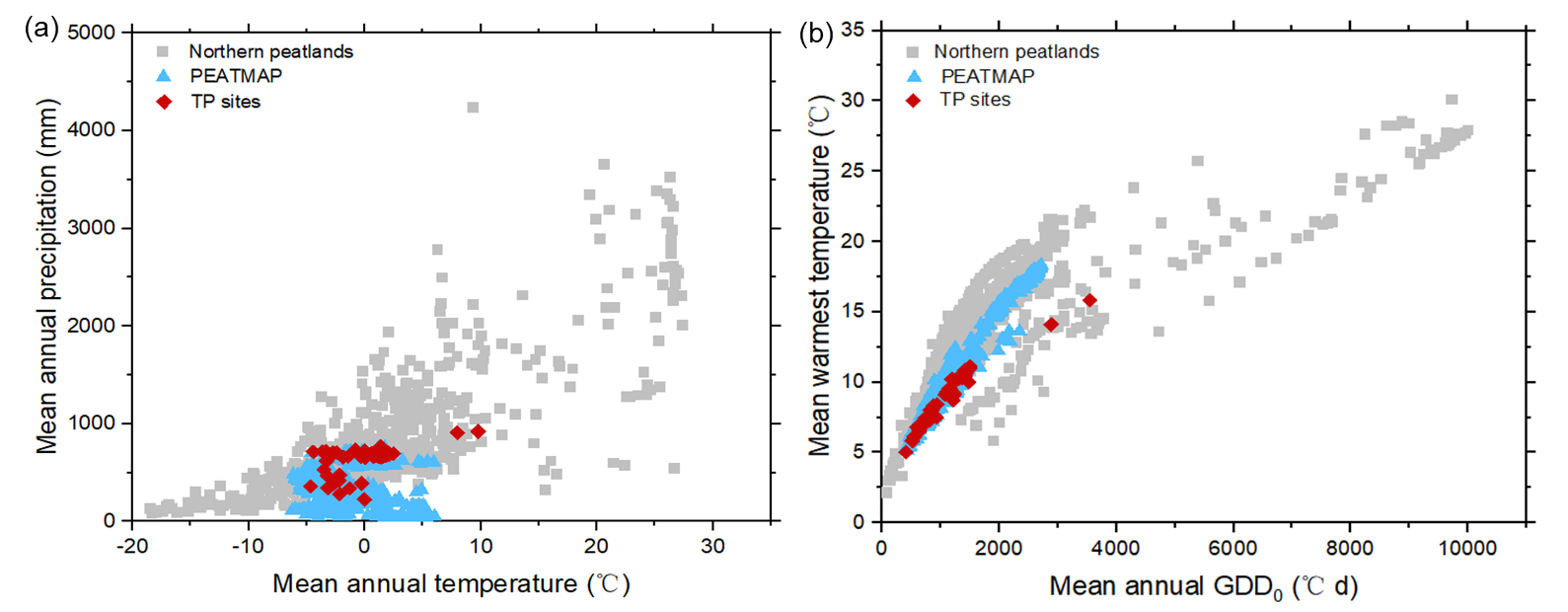


Supplementary Figure S7. Comparison of the environmental space between TP peatlands and northern peatlands (data from Yu et al. 2009 and Morris et al., 2018).


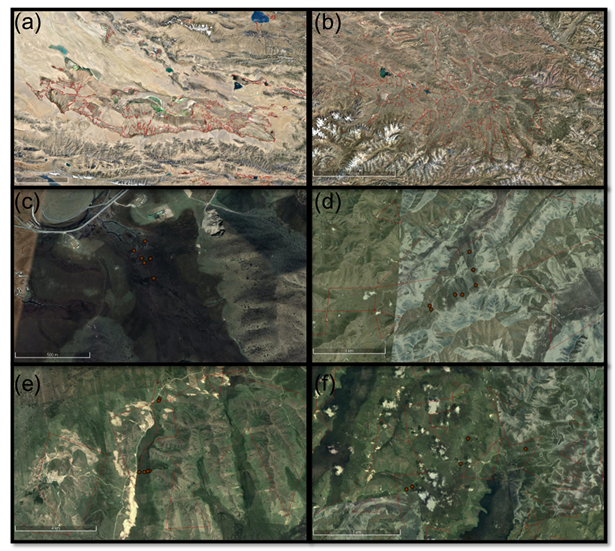


Supplementary Figure S8. Comparisons of Google Earth images and PEATMAP. (a) The peatland distribution (red polygons) from PEATMAP in the Qaidam Basin in the northern TP and (b) in Three-River-Source National Park. The peatland sites that are not captured by PEATMAP include: (c) Hongyuan2, Hongyuan3, ZB10-C8, ZB10-C9, ZB10-BD11, ZB10-BD12, (d) ZB08-C1, ZB10-BD1, ZB10-BD40, ZB10-BD14, ZB10-BD42,Tangke, Hongyuan, (e) ZB10-BD28, ZB10-BD29, ZB10-BD30, ZB10-BD31, and (f) ZB10-BD43, ZB10-BD48, Denahequ S, Denahe1.


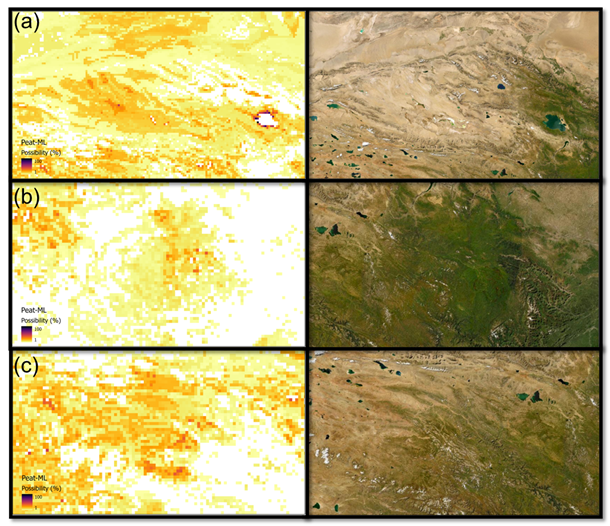


Supplementary Figure S9. Comparison between Google Earth images (right) and the Peat-ML map (left, Melton et al., 2022). (a), (b) and (c) are located in northern TP, Zoige peatland complex, and centre TP, respectively.


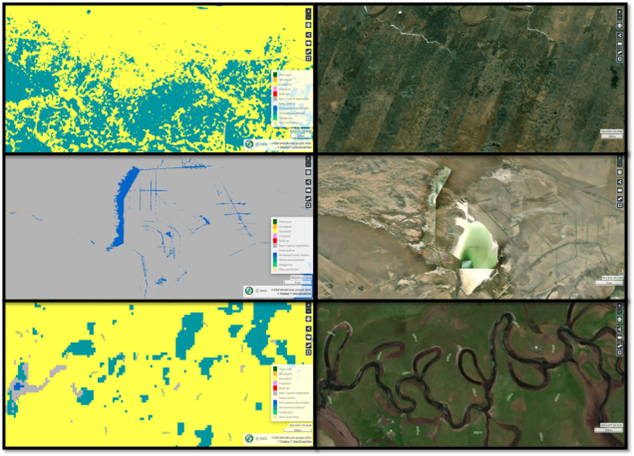


Supplementary Figure S10. Comparison between Google Earth images (right) and the Worldwide land cover mapping (left) (https://esa-worldcover.org/en); (a) Zoige peatland complex region, where grassland and herbaceous wetlands are not always well captured by the ESA map, (b) Qaidam Basin, where the lake does not appear on the worldwide map, and (c) Zoige peatland complex region, where the river is labelled as grassland in the worldwide map.


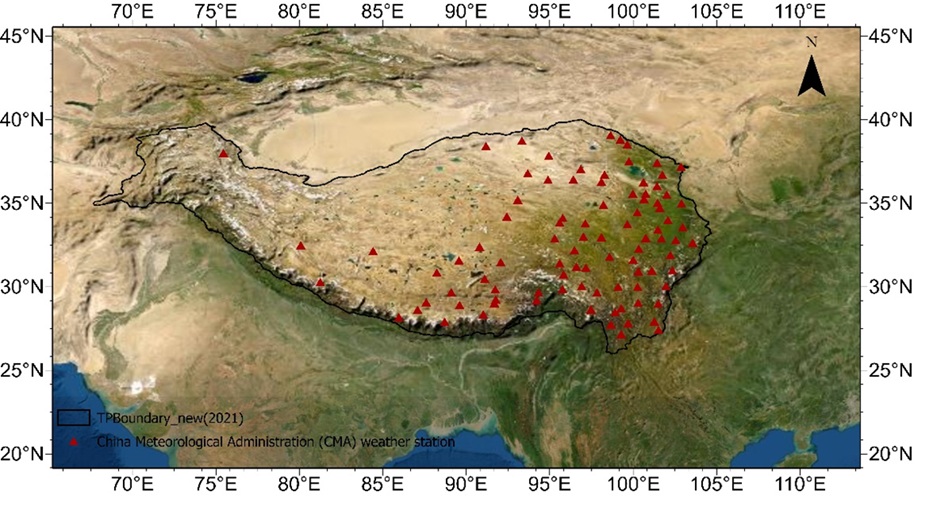


Supplementary Figure S11. Distribution of the TP Meteorological Administration (CMA) weather stations ([http://data.cma.cn](http://data.cma.cn/)).


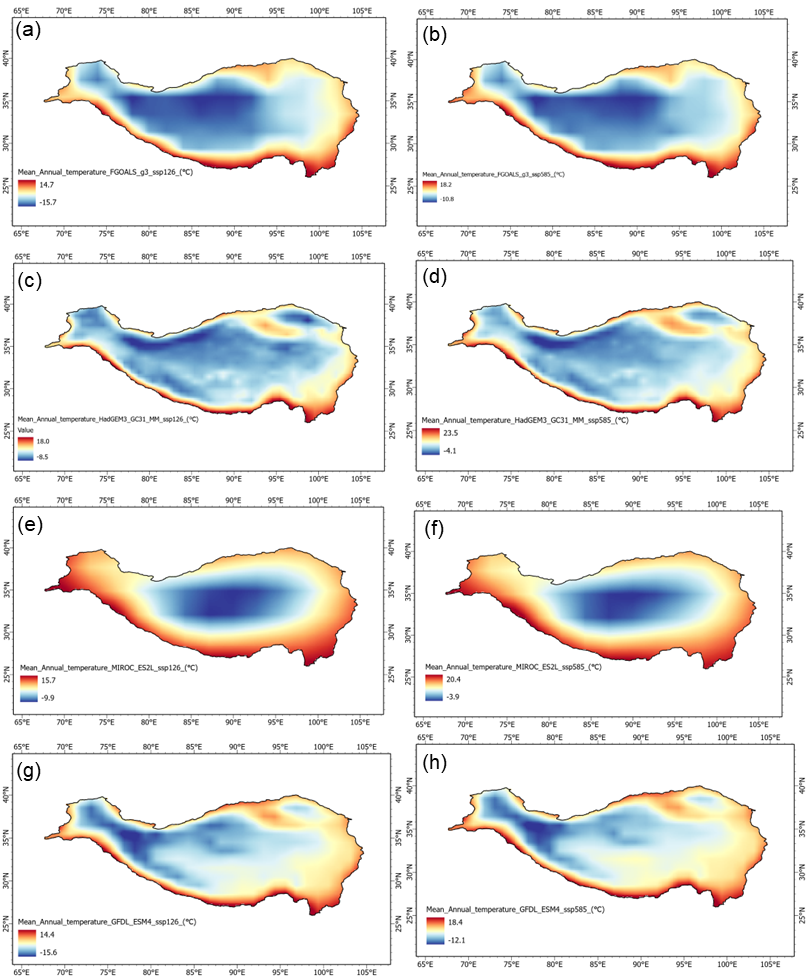


Supplementary Figure S12. Mean annual temperature changes for the 2100 AD compared to the modern baseline period (CRU CL v. 2.0, 1961-1990) on the TP, for four different model outputs (FGOALS_g3, HadGEM3_MM, MIROC_ES2L and GFDL_ESM4) under scenarios of low-to-high warming (Shared Socioeconomic Pathway (SSP) 1-2.6 and SSP5-8.5).


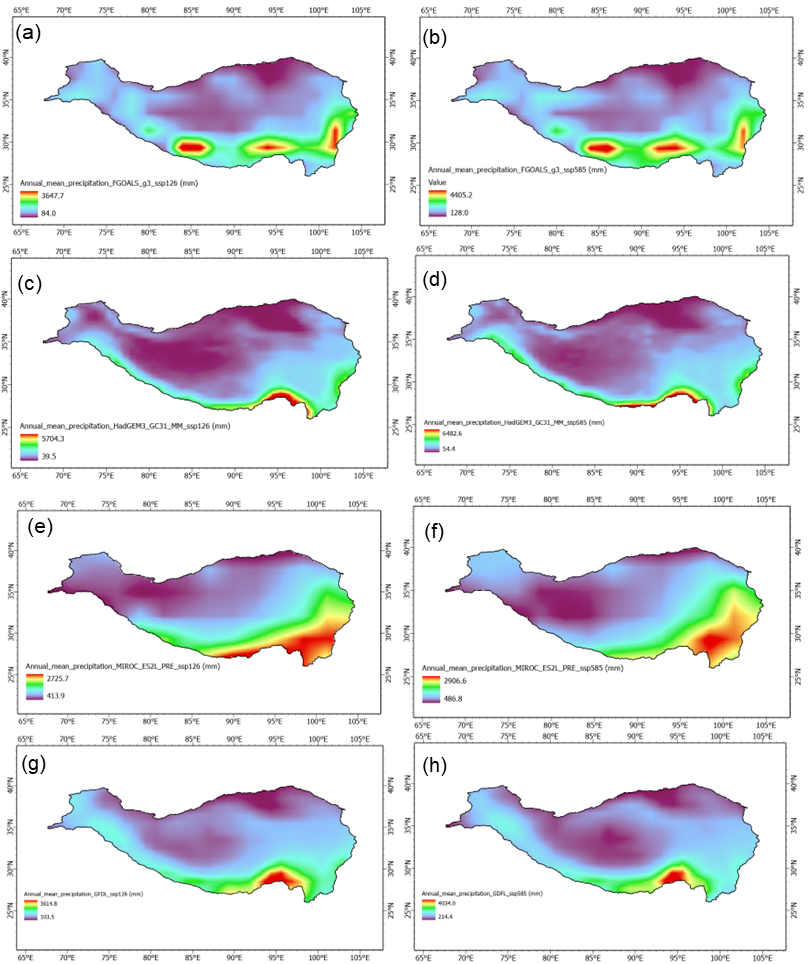


Supplementary Figure S13. Mean annual precipitation changes for the 2100 AD compared to the modern baseline period (CRU CL v. 2.0, 1961-1990) on the TP, for four different model outputs (FGOALS_g3, HadGEM3_MM, MIROC_ES2L and GFDL_ESM4) under scenarios of low-to-high warming (Shared Socioeconomic Pathway (SSP) 1-2.6 and SSP5-8.5).


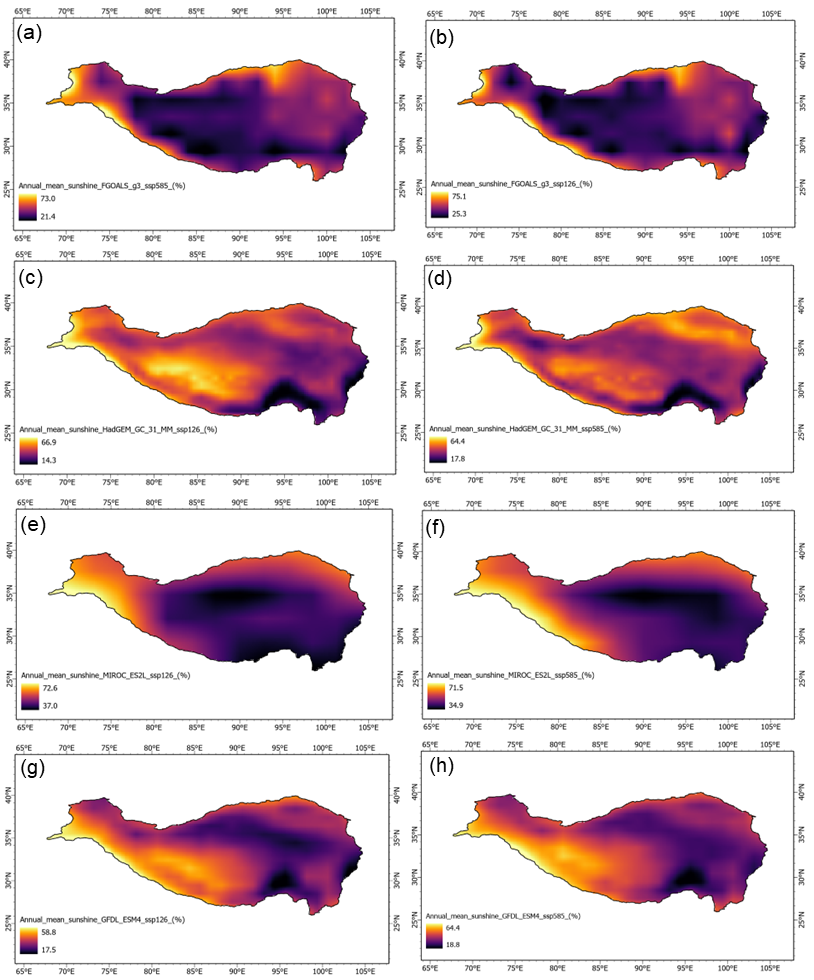


Supplementary Figure S14. Mean annual sunshine changes for the 2100 AD compared to the modern baseline period (CRU CL v. 2.0, 1961-1990) on the TP, for four different model outputs (FGOALS_g3, HadGEM3_MM, MIROC_ES2L and GFDL_ESM4) under scenarios of low-to-high warming (Shared Socioeconomic Pathway (SSP) 1-2.6 and SSP5-8.5).
